# Merging Boron and Carbonyl based MR‐TADF Emitter Designs to Achieve High Performance Pure Blue OLEDs**

**DOI:** 10.1002/anie.202305182

**Published:** 2023-06-05

**Authors:** Sen Wu, Le Zhang, Jingxiang Wang, Abhishek Kumar Gupta, Ifor D. W. Samuel, Eli Zysman‐Colman

**Affiliations:** ^1^ Organic Semiconductor Centre EaStCHEM School of Chemistry University of St Andrews St Andrews Fife KY16 9ST UK; ^2^ Organic Semiconductor Centre SUPA School of Physics and Astronomy University of St Andrews St Andrews KY169SS UK

**Keywords:** Blue Emission, Boron, Multiresonant Thermally Activated Delayed Fluorescence, Organic Light-Emitting Diodes

## Abstract

Multiresonant thermally activated delayed fluorescence (MR‐TADF) compounds are attractive as emitters for organic light‐emitting diodes (OLEDs) as they can simultaneously harvest both singlet and triplet excitons to produce light in the device and show very narrow emission spectra, which translates to excellent color purity. Here, we report the first example of an MR‐TADF emitter (DOBDiKTa) that fuses together fragments from the two major classes of MR‐TADF compounds, those containing boron (DOBNA) and those containing carbonyl groups (DiKTa) as acceptor fragments in the MR‐TADF skeleton. The resulting molecular design, this compound shows desirable narrowband pure blue emission and efficient TADF character. The co‐host OLED with DOBDiKTa as the emitter showed a maximum external quantum efficiency (EQE_max_) of 17.4 %, an efficiency roll‐off of 32 % at 100 cd m^−2^, and Commission Internationale de l’Éclairage (CIE) coordinates of (0.14, 0.12). Compared to DOBNA and DiKTa, DOBDiKTa shows higher device efficiency with reduced efficiency roll‐off while maintaining a high color purity, which demonstrates the promise of the proposed molecular design.

## Graphical abstract

The multiresonant thermally activated delayed fluorescence (MR‐TADF) emitter, DOBDiKTa merges the motifs of boron‐based and ketone‐based MR‐TADF emitters. Using this molecular design, this compound shows deep blue, narrowband emission. The co‐host device with DOBDiKTa achieved a high maximum external quantum efficiency (EQE_max_) of 17.4 % and blue emission with Commission Internationale de l’Éclairage (CIE) coordinates of (0.14, 0.12).

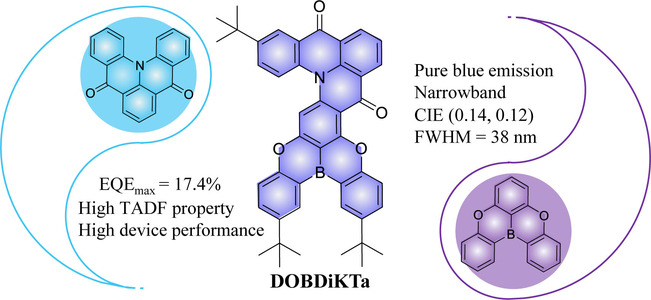

## Introduction

Thermally activated delayed fluorescence (TADF) materials show great potential as emitters in the organic light‐emitting diodes (OLEDs),[^1^, ^2^, ^3^] due to their capacity to harvest both singlet and triplet excitons and convert these into light. This is possible as a result of fast reverse intersystem crossing (RISC), which can occur in materials with small singlet‐triplet energy gaps (Δ*E*_ST_)[^4^, ^5^] and outcompetes non‐radiative decay pathways. In traditional donor‐acceptor (D‐A) TADF compounds, the small Δ*E*_ST_ results from the small exchange integral of the frontier orbitals that is possible in strongly twisted conformations where the donor and acceptor are mostly electronically decoupled.^[6]^ A consequence of this molecular design that leads to long‐range charge transfer (LRCT) excited states is that the emission spectrum is broad, characterized by a full width at half maximum (FWHM) larger than 70 nm.^[7]^ Thus, OLEDs using D‐A TADF emitters typically show poor color purity. Notably, to obtain the blue electroluminescence (EL) that can meet the BT.2020 standard, i.e., CIEy≤0.05, without significant loss of efficiency caused by the use of color filters or microcavities will require the use of narrowband emitters.[^8^, ^9^]

In 2016, Hatakeyama and co‐workers reported the first examples of boron‐based multiresonant TADF (MR‐TADF) emitters, which showed simultaneously high photoluminescence quantum yields (Φ_PL_), sufficiently small Δ*E*_ST_, and narrowband blue emission (FWHM of 28 nm).^[10]^ This seminal work has catalyzed significant efforts towards the development of boron‐containing analogues, and also led to the expansion of the chemical space of MR‐TADF emitters towards derivatives with the p‐dopant replaced by carbonyl moieties.[^11^, ^12^, ^13^] The oxygen‐bridged boron‐contained MR‐TADF emitter DABOA was the first p‐, and n‐doped polycyclic aromatic hydrocarbon reported by Hatakeyama.[^14^, ^15^] This compound shows near‐UV narrow emission, with a *λ*_PL_ of 398 nm and a FWHM of 34 nm in toluene solution.[^15^, ^16^, ^17^] Since this first report in 2015, many derivatives of DABOA (aka DOBNA) have been reported, where DOBNA frequently takes the role of a weak acceptor in donor‐acceptor TADF emitters. In 2019, both Liao's group and our group reported some of the first examples of ketone/nitrogen‐based MR‐TADF emitters (DiKTa, aka QAO), and the OLEDs achieved an EQE_max_ of 14.7 %, with *λ*_EL_ of 465 nm and FWHM of 39 nm.[^11^, ^18^] Numerous derivatives of DiKTa have since been reported, with the corresponding OLEDs achieving EQE_max_ in some examples of greater than 30 %.[^11^, ^18^, ^19^, ^20^, ^21^, ^22^] Despite these impressive advances, devices that can produce the desired pure blue emission (CIE_*x*_+CIE_*y*_<0.3) remain rare.

**Figure 1:**
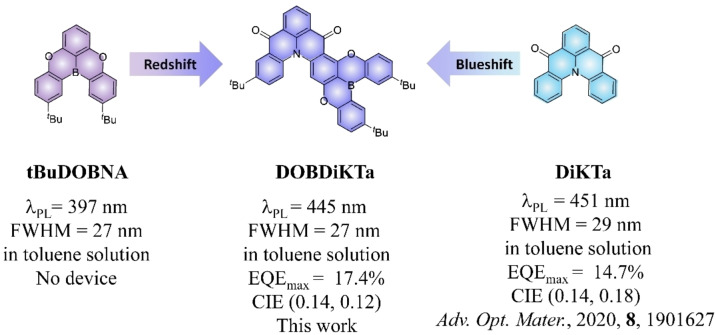
Comparison of the properties and structures of **tBuDOBNA**, **DOBDiKTa**, and **DiKTa**.

Here, we present a high‐performance pure blue MR‐TADF emitter design strategy by fusing boron and carbonyl MR‐TADF cores where the boron and nitrogen atoms are disposed *para* to each other in a B‐π‐N manner. By fusing the two MR‐TADF emitters of DOBNA and DiKTa into a single molecule, DOBDiKTa, not only are the MR‐TADF properties conserved, but a desired color tuning to the blue and improved RISC rates are also achieved. As shown in Figure 1, the boron atom is embedded *para* to the nitrogen atom of DiKTa, thus the emission is blue‐shifted to 445 nm between DiKTa (*λ*_PL_=451 nm) and tBuDOBNA (*λ*_PL_=397 nm) in toluene. The emission of 1.5 wt % doped film of DOBDiKTa in a 1 : 1 mixture of 1,3‐bis(*N*‐carbazolyl)benzene :2,8‐bis(diphenylphosphoryl)‐dibenzo[b,d]thiophene (mCP:PPT) as a co‐host system is 461 nm (FWHM of 38 nm), which is nearly identical to the emission observed in mCP at the same doping concentration (*λ*_PL_=460 nm with FWHM of 37 nm). Compared with DiKTa, OLEDs using the mCP:PPT co‐host with DOBDiKTa as the emitter show an increased EQE_max_ of 17.4 % and bluer emission at *λ*_EL_ of 458 nm (FWHM of 38 nm) associated with improved CIE coordinates of (0.14, 0.12).

## Results and Discussion

DOBDiKTa was synthesized via a three‐step linear reaction (Scheme S1). DOBNPh was obtained in good yield through a palladium‐catalysed Buchwald–Hartwig coupling between DOBBr and 4‐*tert*‐butylaniline. DOBNPh was synthesized via a copper‐catalysed Ullman coupling between DOBNPh and 2‐bromoisophthalic acid dimethyl ester. Quantitative saponification followed by a two‐fold intramolecular Friedel–Crafts acylation of the in situ‐prepared acylchloride derivative in the presence of the Lewis acid SnCl_2_ afforded the target DOBDiKTa in a moderate yield of 51 %. The identity and purity of DOBDiKTa were determined using a combination of ^1^H and ^13^C NMR spectroscopy, melting point determination, high‐resolution mass spectrometry, element analysis, and high‐pressure liquid chromatography (HPLC), shown in Figure S1–S11.

**Figure 2:**
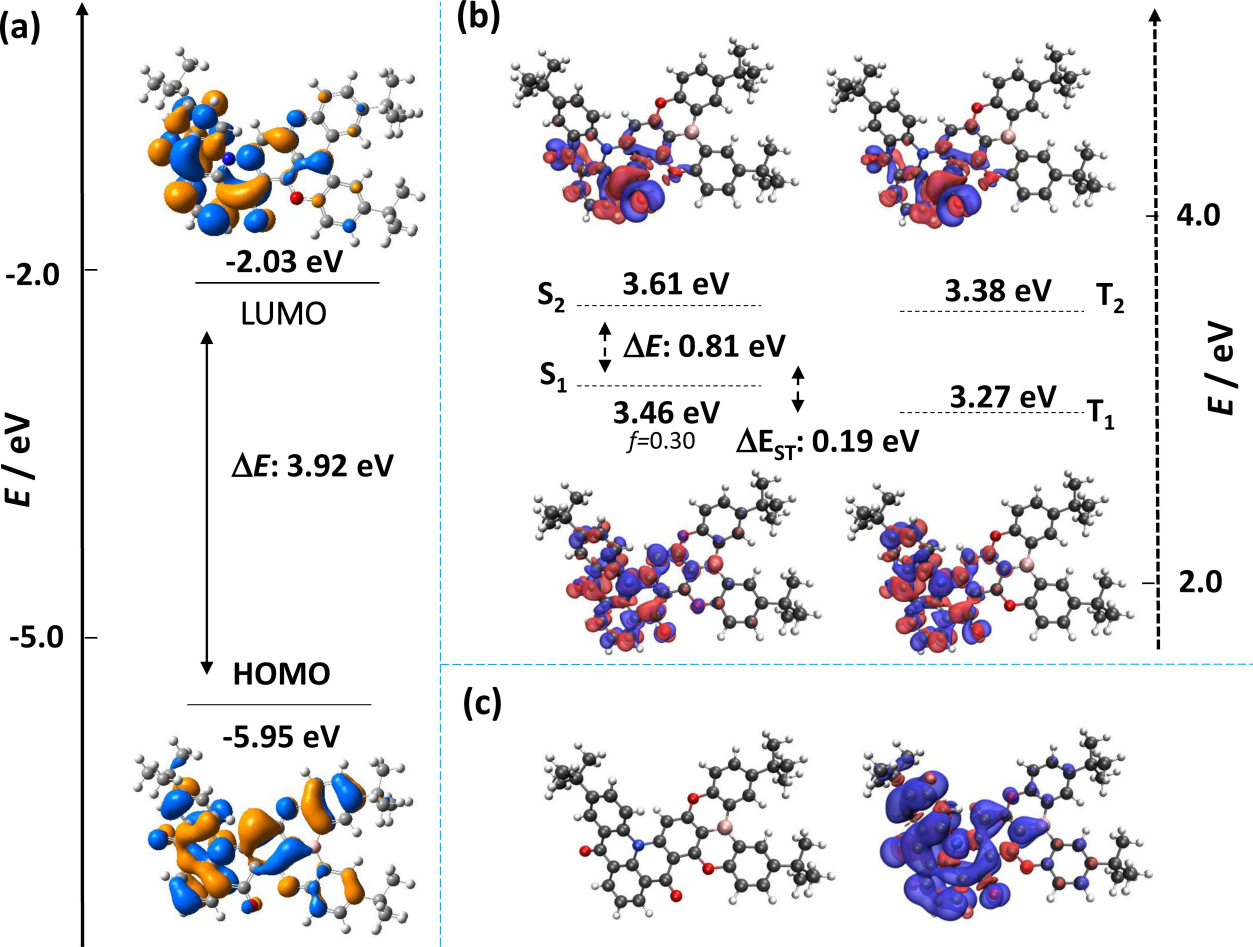
a) Distributions of the frontier molecular orbitals of DOBDiKTa, calculated in the gas phase at the PBE0/6‐31G(d,p) level. b) Difference density plots of S_1_/S_2_ and T_1_/T_2_ excited states (calculated in the gas phase at the SCS‐ADC(2)/cc‐pVDZ level) for DOBDiKTa. *f* is the oscillator strength. c) Triplet spin density of DOBDiKTa calculated in the gas phase at the T_1_ optimized geometry at the uPBE0/6‐31G(d,p) level.

The molecular design for DOBDiKTa was rationalized through a ground‐ and excited‐state properties computational study using a combination of gas‐phase DFT (PBE0/6‐31G(d,p)) and wavefunction‐based (SCS‐ADC(2)/cc‐pVDZ) methods, respectively (Figure 2); indeed, we have previously demonstrated DFT methods are not suitable for accurately modelling the excited states of MR‐TADF compounds and so a higher level of theory is required.[^23^, ^24^] From Figure 2a, the highest occupied molecular orbitals (HOMO) is distributed over the entire π‐conjugated system and the lowest unoccupied molecular orbitals (LUMO) is mostly localized on the DiKTa fragment, similar to that calculated for DiKTa (Figure S12). The calculated HOMO and LUMO levels are −5.95 and −2.03 eV, respectively, which are destabilized compared to those of DiKTa (HOMO: −6.20 eV and LUMO: −2.23 eV) and stabilized compared to those of DOBNA (HOMO: −5.68 eV and LUMO: −1.71 eV). In the S_1_ and T_1_ excited states, the electron density is localized on the DiKTa moiety (Figure 2b) and the corresponding difference density plots also reveal the expected alternating increasing and decreasing electron density pattern that is characteristic of excited states of short‐range charge transfer (SRCT) character. The calculated S_1_ and T_1_ energies of DOBDiKTa are 3.46 and 3.27 eV, respectively, which are almost the same as those of DiKTa (S_1_=3.46 eV and T_1_=3.20 eV, Figure S12) and a modestly stabilized to those of DOBANA of (S_1_=3.65 eV and T_1_=3.44 eV, Figure S13), indicating that a similar emission energy to DiKTa is expected. The Δ*E*_ST_ of DOBDiKTa is 0.19 eV, which is smaller than both of DiKTa (0.26 eV) and DOBNA (0.20 eV). The oscillator strength for the S_0_‐S_1_ transition in DOBDiKTa is larger at 0.30 than in both DiKTa (0.20) and DOBNA (0.17), implying a higher Φ_PL_ in the former. DOBDiKTa shows a small structural relaxation at the optimized singlet excited‐state geometry compared to the ground‐state geometry (Figure S14), which suggests that this compound should exhibit very narrowband emission. The spin‐density distribution (SDD) of the T_1_ state of DOBDiKTa is localized on the DiKTa fragment. The benefit of the carbonyl groups in DOBDiKTa is evidenced by the much larger SOC matrix element (SOCME) of 1.64 cm^−1^ compared with that of DOBNA of 0.006 cm^−1^ (SOCME of DiKTa is 1.83 cm^−1^) between S_1_ and T_1_ (Figure S15)_._ There are additionally five intermediate triplet states that lie between S_1_ and T_1_. The SOCME values range from 1.88 to 4.97 cm^−1^, which are of intermediate values to those of DiKTa (⟨S_1_|*Ĥ*_SOC_|T_2_⟩=6.32 cm^−1^, ⟨S_1_|*Ĥ*_SOC_|T_3_⟩=7.77 cm^−1^, ⟨S_1_|*Ĥ*_SOC_|T_4_⟩=7.59 cm^−1^) and DOBNA (⟨S_1_|*Ĥ*_SOC_|T_2_⟩=0.45 cm^−1^ and ⟨S_1_|*Ĥ*_SOC_|T_3_⟩=0.08 cm^−1^ ), and point to a RISC mechanism via spin‐vibronic coupling involving one or more of these states.

**Figure 3:**
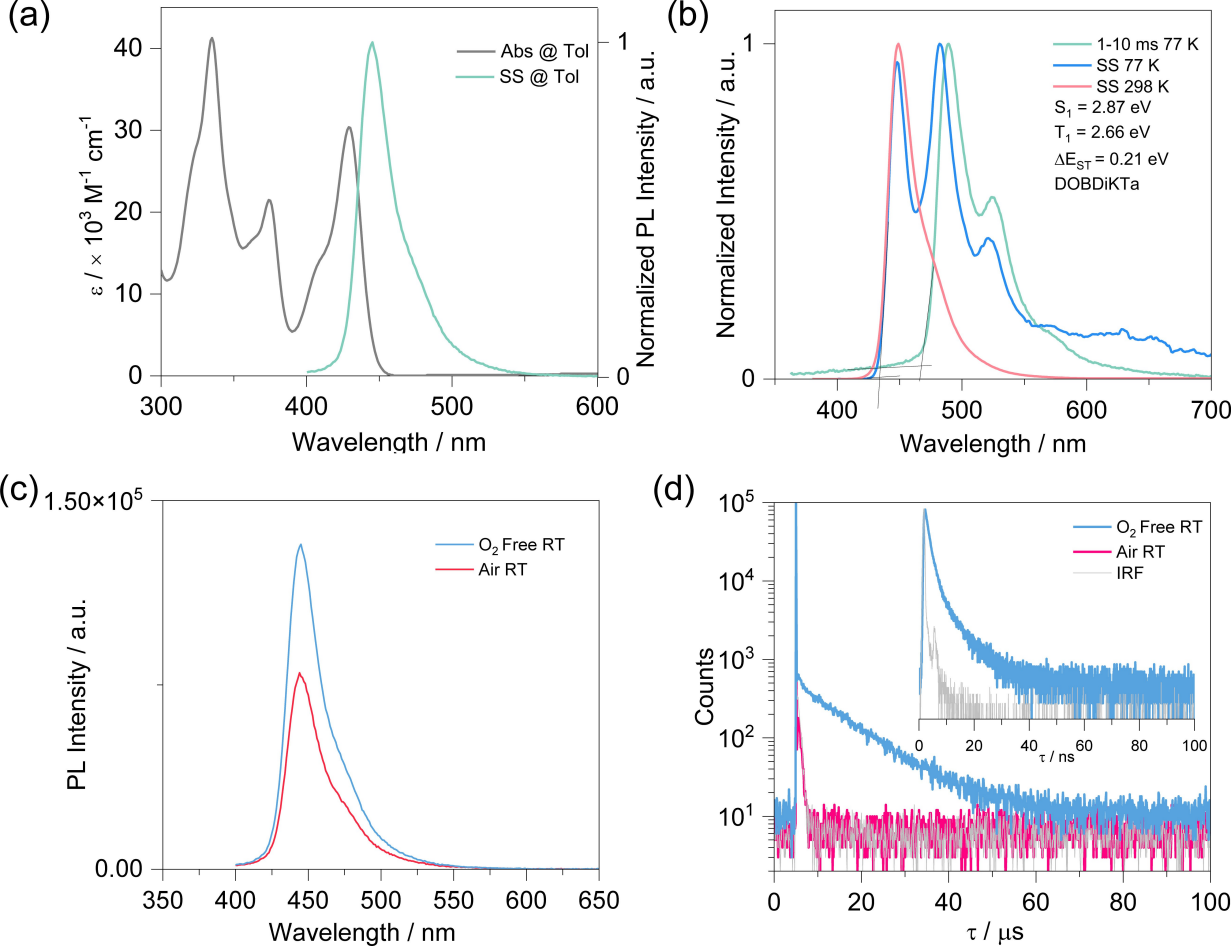
a) Absorption and steady‐state PL spectra (SS) in toluene at room temperature (*λ*_exc_=340 nm); b) prompt PL and delayed emission spectra measured in toluene glass at 77 K (*λ*_exc_=340 nm); c) Comparison of the intensity of the PL spectra in both aerated and degassed toluene solutions (*λ*_exc_=340 nm); d) Time‐resolved PL decay (*λ*_exc_=375 nm) in toluene solution (inset Figure is the PL decay of the prompt component). RT = room temperature.

The energies of the frontier molecular orbitals were inferred from the electrochemistry, measured by cyclic voltammetry (CV) and differential pulse voltammetry (DPV) in degassed DMF. The electrochemical data reported versus SCE are summarized in Table S1. As shown in Figure S17, there is an irreversible oxidation and a quasi‐reversible reduction wave, with associated peak potentials from the DPV of 1.47 and −1.50 eV, respectively. The corresponding HOMO and LUMO levels are −5.78 and −2.80 eV, respectively, leading to an intermediate HOMO–LUMO gap of 2.98 eV compared to those tBuDOBNA (3.00 eV) and tBuDiKTa (2.85 eV).^[25]^ The room temperature UV/Visible absorption, steady‐state photoluminescence (PL) and 77 K phosphorescence spectra of DOBDiKTa in dilute toluene solution are depicted in Figure 3. The absorption spectrum possesses three distinguished peaks at 335, 374 and 429 nm (Figure 3a). The absorption band at 300–350 nm is assigned to π‐π^*^ transitions over the whole skeleton. The absorption band at 374 nm is assigned to the SRCT transition localized on the tBuDOBNA unit of DOBDiKTa given its similar energy to that found the absorption spectrum of tBuDOBNA (383 nm) as shown in Figure S18. The lowest energy absorption centered at 429 nm is the SRCT transition on the DiKTa fragment (Figure S16), according to the similar energy of this band to that of DiKTa (433 nm). Thus, though the two MR‐TADF fragments of tBuDOBNA and DiKTa are annulated in DOBDiKTa, in the ground state absorption spectra, they act essentially as independent chromophores. The steady‐state PL of DOBDiKTa in toluene evidences a narrowband blue emission, with *λ*_PL_ of 445 nm and a full width at half maximum (FWHM) of 27 nm. This emission is in between that of tBuDOBNA (397 nm) and DiKTa (451 nm). The mirrored absorption and emission spectra, the small Stokes shift of 16 nm (0.12 eV, shown in Figure S19) and small FWHM all reflect the small degree of geometric reorganization in the excited state, which is confirmed by the small configuration difference between S_1_ and S_0_ shown in Figure S14. The change in PL as a function of solvent polarity (Figure S21) reflects a modest positive solvatochromism that is consistent with an emissive excited state of SRCT character that is a hallmark of MR‐TADF emitters. The energies of the S_1_ and T_1_ states were determined from the onsets of the prompt fluorescence and delayed emission in toluene glass at 77 K (Figure 3b). The calculated S_1_ and T_1_ energies are 2.87 and 2.66 eV, respectively, revealing a modest Δ*E*_ST_ of 0.21 eV, which is in between those of DiKTa (0.22 eV) and tBuDOBNA (0.20 eV) (Figure S20). Steady‐state and time‐resolved PL in both aerated and degassed in toluene are shown in Figures 3c and 3d. The PL spectrum is somewhat quenched in air with the photoluminescence quantum yield, Φ_PL_, decreasing from 48 % to 29 %. The transient decays show a quenching of the delayed emission in air. The prompt and delayed lifetimes, τ_p_ and τ_d_, are 1.9 ns and 11 μs in degassed toluene solution; the delayed emission is not detectable in aerated solution. Rate constants for the various radiative and non‐radiative processes are summarized in Table S2. The rate constant for RISC, *k*_RISC_, is 8.4×10^4^ s^−1^_._ All these results imply contributions from triplet excitons. By contrast, delayed emission was not observed in toluene solutions of DiKTa and tBuDOBNA. The photophysical properties are summarized in Table 1.

**Table 1:** Photophysical data of DOBDiKTa, tBuDOBNA and DiKTa.

Emitters		*λ*_abs_ [nm]	Φ_PL_ [%]^[c]^	*λ*_PL_ [nm]^[d]^	FWHM [nm]^[e]^	S_1_ [eV]^[f]^	T_1_ [eV]^[g]^	Δ*E*_ST_/ [eV]^[h]^	τ_p_ [ns]^[i]^	τ_d_ [μs]^[i]^
DOB‐DiKTa	sol^[a]^	430	48	445	27	2.87	2.66	0.21	1.9	11
	film^[b]^	N/A	75	460	37	2.77	2.60	0.17	2.6	43
tBuDOBNA	sol^[a]^	383	N/A	397	26	3.26	3.06	0.20	N/A	N/A
	film^[b]^	N/A	62	407	34	N/A	N/A	N/A	9.8	–
DiKTa	sol^[a]^	433	N/A	451	28	2.81	2.59	0.22	N/A	N/A
	film^[b]^	N/A	46	467	46	N/A	N/A	N/A	4.8	242

[a] Measured in toluene solution at a concentration of 1×10^−5^ M; [b] Measured in spin‐coated 1.5 wt % doped thin films in mCP. [c] the Φ_PL_ of the solution was measured using the optically dilute method and of the film was measured using an integrating sphere under nitrogen (*λ*_exc_=340 nm).^[26]^ [d] Obtained at 298 K, *λ*_exc_=340 nm. [e] Full‐width at half‐maximum; [f] Obtained from the onset of the SS PL spectrum at 77 K. [g] Obtained from the onset of the delayed emission spectrum (1–10 ms) at 77 K (*λ*_exc_=340 nm). [h] Δ*E*_ST_=*E*(S_1_)−*E*(T_1_). [i] Measured at 298 K under vacuum, *λ*_exc_=379 nm.

**Figure 4:**
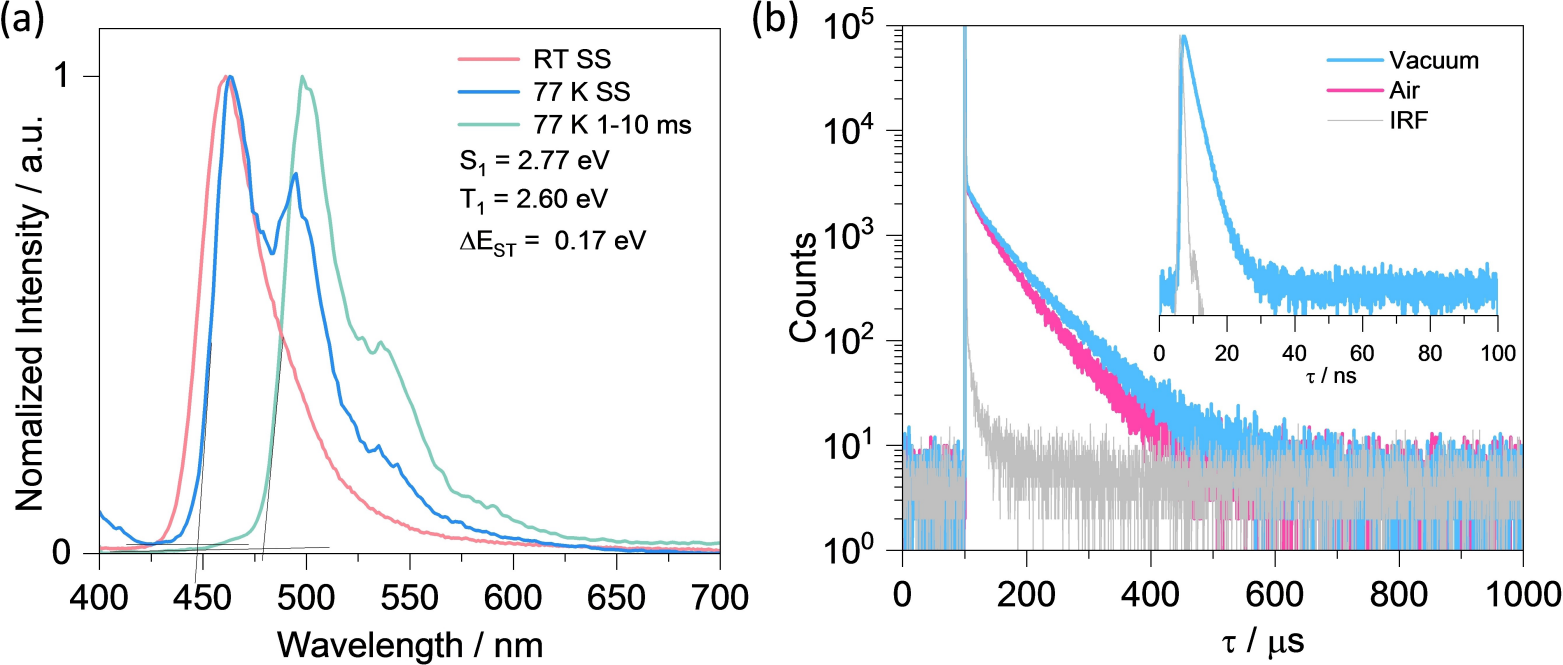
a) Steady‐state photoluminescence spectra of **DOBDiKTa** at room temperature and at 77 K, phosphorescence spectra measured at 77 K (1–10 ms) in 1.5 wt % doped film in mCP (*λ*_exc_=340 nm); and b) TRPL decays (*λ*_exc_=379 nm) in 1.5 wt % doped film in mCP (inset figure is the PL decay of the prompt component).

We next investigated the photophysical properties of DOBDiKTa in doped films in a host of suitably high triplet energy (E_T1_=2.81 eV), mCP as this was the host used in our prior studies of DiKTa.[^22^, ^27^] The emission in mCP doped film red‐shifts with increasing doping concentration from 1.5 to 10 wt % (Figure S23a), reflecting increasing contributions from aggregates to the emission. The Φ_PL_ likewise drops from 75 % to 47 % with increasing doping concentration (Figure S23b). Thus, 1.5 wt % doped film in mCP were used for the solid‐state photophysical study as shown in Figure 4. The steady‐state PL shows narrowband blue emission at *λ*_PL_ of 460 nm with FWHM of 37 nm (Figure 4a), which is red‐shift and broader compared to the spectrum in dilute toluene solution (*λ*_PL_=445 nm, FWHM=27 nm). These spectral changes can be attributed to the presence of aggregates as well as host–guest interactions. The emission is also of intermediate wavelength to those of DiKTa (467 nm) and tBuDOBNA (407 nm), as shown in Figure S24. The S_1_ energy was inferred from the high‐energy onset of the steady‐state PL at 77 K to be 2.77 eV while the T_1_ energy was determined from the onset of the time‐gated phosphorescence at 77 K to be 2.60 eV; thus, the Δ*E*_ST_ is 0.17 eV (Figure 4). The S_1_ and T_1_ levels are both stabilized compared with those measured in toluene, resulting in a smaller Δ*E*_ST_, which can be attributed to a combination of increased polarity of the host and possibly the presence of aggregates and host–guest interactions. The PL spectrum is somewhat quenched in air, with Φ_PL_ decreasing from 75 % to 67 % (Figure S22). The transient PL decay under vacuum shows biexponential kinetics with a τ_p_ of 2.6 ns and a τ_d_ of 43 μs (Figure 4b). By contrast, DiKTa shows prompt and delayed lifetimes of 4.8 ns and 242 μs, while tBuDOBNA only shows a prompt lifetime of 9.8 ns (Figure S26). The singlet radiative transition rate constant (*k* ^S^_r_) of 6.9×10^7^ s^−1^ is slower than rate constants of intersystem crossing (*k*_ISC_=3.2×10^8^ s^−1^), which is due to the strong spin‐orbital coupling (SOC) induced by the carbonyl groups.^[19]^ The rate constant for RISC, *k*_RISC_, at 8.8×10^4^ s^−1^ is almost 3.5‐fold faster than that of DiKTa (*k*_RISC_, at 2.5×10^4^ s^−1^). The intensity of the delayed PL increases with increasing temperature, evidencing the TADF character of this compound (Figure S25). All these results indicate that DOBDiKTa has an improved TADF character compared to DiKTa.

**Figure 5:**
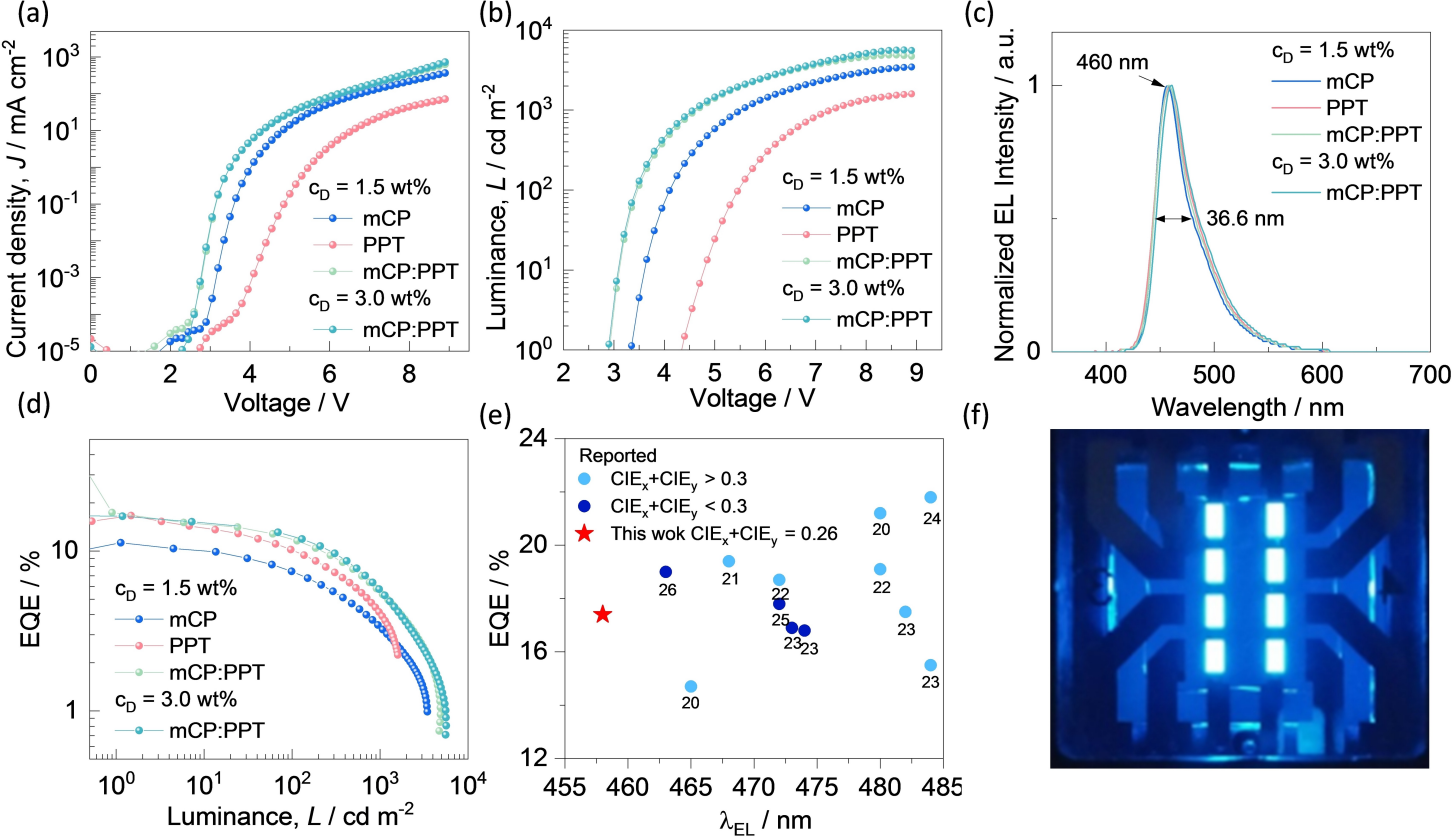
a) Current density versus voltage characteristics of the devices; b) Luminance versus voltage characteristics for the devices; c) Electroluminescence spectra of the devices; d) External quantum efficiency versus luminance curves for the devices; e) EQE_max_ of reported ketone‐based MR‐TADF OLEDs as a function of *λ*_EL_; f) Photograph of the devices.

Finally, we fabricated evaporated OLEDs with the following device stack: ITO/HATCN (5 nm)/TAPC (40 nm)/TCTA (10 nm)/mCP (10 nm)/EML (20 nm)/PPT (10 nm)/TmPyPB (50 nm)/LiF (0.8 nm)/Al (100 nm), where indium tin oxide (ITO) is the anode, 1,4,5,8,9,11‐hexaazatriphenylenehexacarbonitrile (HATCN) is the hole inject layer, 4,4′‐cyclohexylidenebis[*N,N*‐bis(4‐methylphenyl)benzenamine] (TAPC) and tris(4‐carbazoyl‐9‐ylphenyl)amine (TCTA) act as hole‐transport layers, respectively. mCP is used to block excitons, 1,3,5‐tri(*m*‐pyridin‐3‐ ylphenyl)benzene (TmPyPB) acts as the electron‐transporting material, and LiF modifies the work function of the aluminum cathode. The device stack and the chemical structures of the materials applied in devices are shown in Figure S29. The device performance is summarized in Figures 5 and S31 and the data are collected in Table 2.

**Table 2:** Electroluminescence data.

Device	*V*_on_ [V]	*λ*_EL_ [nm]	FWHM [nm]	CIE (*x*,*y*)	*L*_max_ [cd m^−2^]	EQE_max/100/1000_ [%]
Device 1^[a]^	3.3	457	36.8	0.14,0.11	3454	11.3/7.4/3.2
Device 2^[b]^	4.3	459	41.4	0.14,0.12	1593	16.7/10.2/4.0
Device 3^[c]^	2.9	458	38.1	0.14,0.12	4707	17.4/11.8/5.5
Device 4^[d]^	2.9	460	39.5	0.14,0.13	5574	16.5/12.4/5.7

Emissive layer [a] 1.5 wt % DOBDiKTa in mCP; [b] 1.5 wt % DOBDiKTa in PPT; [c] 1.5 wt % DOBDiKTa in 1 : 1 mCP : PPT; [d] 3 wt % DOBDiKTa in 1 : 1 mCP : PPT.

Firstly, we fabricated devices using mCP as the host, device 1. The electroluminescence (EL) spectrum shows narrowband blue emission (FWHM=37 nm) at 457 nm (Figure 5c), which was consistent with the thin film PL spectrum (Figure S30). The maximum external quantum efficiency (EQE_max_) was 11.3 % (Figure 5d), much lower than the theoretical prediction of 15 % according to EQE=IQE×*η*_out_, where IQE is 75 % and the light out‐coupling efficiency (*η*_out_) is assumed to be 20 %.^[28]^ To exclude the possible exciton loss due to the insufficient exciton confinement of the mCP host, a higher triplet energy level host, PPT [2,8‐bis(diphenylphosphoryl)‐dibenzo[b,d]thiophene, E_T_=2.96 eV] was employed.^[29]^ The Φ_PL_ of the 1.5 wt % doped DOBDiKTa films in PPT increased to 79 % compared to 75 % in mCP at the same doping concentration. Nevertheless, the device (device 2) with the same device structure but using PPT as the host showed EQE_max_ as high as 16.7 %, which is almost a 50 % improvement compared to device 1. The significant improvement cannot be explained by the photoluminescence quantum yield alone, but instead should be related to the host material and bipolar carrier balance in the emitting layer (EML). Although a high EQE_max_ was observed in device 2, this device showed a 1 V larger turn‐on voltage (*V*_on_), lower current density, lower luminance, and larger efficiency roll‐off than that of device 1 (Figure 5). In addition, the EL spectrum of device 2 was slightly red‐shifted (*λ*_EL_=459 nm) and broadened (FWHM=41 nm), undermining the color purity (Table 2). The higher V_on_, lower current density and luminance of device 2 than those of device 1 were attributed to the low carrier mobility of the electron transport type PPT host, which, however, helped enhance the recombination probability of injected electrons and holes, leading to an improved EQE at low current density. As the current density increased, the unipolar PPT host showed more severe carrier imbalance, resulting in a larger efficiency roll‐off than the device with the mCP host, which is a hole‐dominated bipolar host.^[30]^ The larger permanent electronic dipole moment of PPT than mCP is responsible for the spectral red‐shift and broadening.

To combine the benefits from each host, i.e., high EQE in PPT, and large luminance and good color purity in mCP, a co‐host strategy was employed with a 1 : 1 ratio of mCP:PPT, and the same doping level of emitter (device 3). The photophysical properties are shown in Figure S28, and this device shows almost the same photoluminescence with *λ*_PL_ at 461 nm and FWHM of 38 nm as well as similar decay kinetics with τ_p_ of 2.6 ns, τ_d_ of 60 μs. The Φ_PL_ of DOBDiKTa in the 1 : 1 co‐host thin film increased to 86 % (Figure S27). Combining these data, the *k*_RISC_ of the co‐host film is 8.4×10^4^ s^−1^ (Table S2). The corresponding OLED showed an improved EQE_max_ of 17.4 %. Due to the bipolar character of the co‐host, the V_on_ was significantly reduced from 4.3 V to 2.9 eV, a value that is similar to the emitted photon energy. In addition, the EL spectrum of device 3 was similar to that of device 1 with *λ*_EL_ of 458 nm and FWHM of 38 nm, resulting in a pure blue emission with CIE coordinates of (0.14, 0.12). Importantly, besides the high EQE_max_ of 17.4 % and high maximum luminance (*L*_max_) of 4707 cd m^−2^, device 3 showed an improved efficiency roll‐off of 32.2 % at 100 cd m^−2^ compared to devices 1 (34.5 %) and 2 (38.9 %). The improved efficiency roll‐off was attributed to maintaining better charge balance in the co‐host, rather than a change in *k*_RISC_. To further optimize the device, the doping concentration was increased to 3 wt % to improve the bipolar carrier balance (device 4). Despite a slightly reduced EQE_max_ of 16.5 %, which was attributed to the decreased Φ_PL_ of 82 %, device 4 showed a higher *L*_max_ of 5574 cd m^−2^ and better roll‐off (24.8 %) at 100 cd m^−2^. Device 3 arguably represents the best performing OLED of the reported carbonyl‐based pure blue (CIE_*x*_+CIE_*y*_<0.3) MR‐TADF OLEDs (Table S3 and Figure 5e).

## Conclusion

In summary, we have developed a pure blue MR‐TADF emitter (DOBDiKTa) by fusing DiKTa and tBuDOBNA together. The resulting compound emits desirably at an intermediate pure blue emission between the sky blue of DiKTa and the purple of tBuDOBNA. DOBDiKTa shows a high Φ_PL_ of 75 %, moderate Δ*E*_ST_ values of 0.17 eV and shorter delayed lifetimes of 43 μs in 1.5 wt % doped mCP films. OLEDs using this emitter showed efficient performance with EQE_max_ of 11.3 % with pure blue emission with *λ*_EL_ of 457 nm. To further optimize the device, a mixed co‐host of 1 : 1 mCP/PPT was employed where the EQE_max_ increased from 11.3 % to 17.4 %, accompanied by a low *V*_on_ of 2.9 V and an increased *L*_max_ from 1600 from 5600 cd m^−2^ without adversely affecting the color purity. The CIE coordinates of (0.14, 0.12) render these OLEDs as the bluest devices with ketone‐based MR‐TADF emitters.

## Conflict of interest

The authors declare no conflict of interest.

1

## Supporting information

As a service to our authors and readers, this journal provides supporting information supplied by the authors. Such materials are peer reviewed and may be re‐organized for online delivery, but are not copy‐edited or typeset. Technical support issues arising from supporting information (other than missing files) should be addressed to the authors.

Supporting Information

Supporting Information

## Data Availability

The research data supporting this publication can be accessed at https://doi.org/10.17630/5c14bb2d‐836d‐42db‐ab6a‐4d85afa564cc.
