# Supplementary material for: Merging Boron and Carbonyl based MR‐TADF Emitter Designs to Achieve High Performance Pure Blue OLEDs
Source: Angew Chem Int Ed Engl. 2023 Jun 5;62(28):e202305182. doi: 10.1002/anie.202305182 (PMC10952889; doi:10.1002/anie.202305182)
Supplement: Supplementary file 1 — Supporting Information [file ANIE-62-0-s001.pdf]

Angewandte Chemie
Eine Zeitschrift der Gesellschaft Deutscher Chemiker

Supporting Information

Merging Boron and Carbonyl based MR-TADF Emitter Designs to Achieve High Performance Pure Blue OLEDs

S. Wu, L. Zhang, J. Wang, A. Kumar Gupta, I. D. W. Samuel, E. Zysman-Colman**

Merging Boron and Carbonyl based MR-TADF Emitter Designs to Achieve High Performance Deep Blue OLEDs

Sen Wu,[a] *Le Zhang,*[a,b] *Jingxiang Wang,*[a] *Abhishek Kumar Gupta,*[a] *Ifor D. W. Samuel*[b]* *and Eli Zysman-Colman*[a]*

[a]Organic Semiconductor Centre, EaStCHEM School of Chemistry, University of St Andrews, St Andrews, Fife, UK, KY16 9ST, Fax: +44-1334 463808; Tel: +44-1334 463826; E-mail: eli.zysman-colman@st-andrews.ac.uk.

[b]Organic Semiconductor Centre, SUPA School of Physics and Astronomy, University of St Andrews, St Andrews, UK, KY16 9SS.

Table of Contents

General methods **S3**

Experimental section **S7**

Computations **S15**

Photophysical characterization **S18**

Devices **S24**

References **S26**

General methods

General Synthetic Procedures. **DOBBr**, **tBuDOBNA** and **DiKTa** were synthesized according to the literature reported method.[1-3] The other reagents and solvents were obtained from commercial sources and used as received unless otherwise stated. Air-sensitive reactions were done under a nitrogen atmosphere using Schlenk techniques. Dry solvents used in the reaction were obtained from a MBRAUN SPS5 solvent purification system. Flash column chromatography was carried out using silica gel (Silia-P from Silicycle, 60 Å, 40-63 μm). Analytical thin-layer-chromatography (TLC) was performed with silica plates with aluminium backings (250 μm with F-254 indicator). TLC visualization was accomplished by 254/365 nm UV lamp. HPLC was conducted on a Shimadzu LC-40 HPLC system. HPLC traces were performed using a Shim-pack GIST 3μm C18 reverse phase analytical column. ^{1}H and ^{13}C and NMR spectra were recorded on a Bruker Advance spectrometer (400 MHz for ^{1}H and 126 MHz for ^{13}C). The following abbreviations have been used for multiplicity assignments: "s" for singlet, "d" for doublet, "t" for triplet, "m" for multiplet, "dd" for doublet of doublets, "dt" for doublet of triplets. ^{1}H and ^{13}C NMR spectra were referenced to the solvent peaks). Melting points were measured using open-ended capillaries on an Electrothermal 1101D Mel-Temp apparatus and are uncorrected. High-resolution mass spectrometry (HRMS) was performed at University of Edinburgh Mass Spectrometry Facility. Elemental analyses were performed by Dr. Joe Casillo at the University of Edinburgh.

Quantum chemical calculations. The calculations were performed using Density Functional Theory (DFT) within Gaussian 16[4] as well as the second order algebraic diagrammatic construction Spin-Component Scaling (ADC(2)-SCS)[5] method using the Turbomole/7.5 package.[6] For the DFT calculation, the ground state, excited singlet and triplet states were optimized with PBE0[7] functional and the 6-31G(d,p) basis set,[8] and excited-state calculations have been performed using Time-Dependent DFT within the Tamm-Dancoff approximation (TDA-DFT)[9, 10] with the same functional and basis set as for the ground-state geometry optimization in gas phase. The molecular orbitals and ESP distribution were visualized with Gaussview 5.0 software. For the ADC(2) calculation, the ground states was optimized with ADC(2)-SCS method and cc-pVDZ basis set in gas phase based on the geometry calculated by DFT.[11] Vertical excited states were performed on the ground state optimized structure using ADC(2)-SCS method. Difference density plots were used to visualize change in electronic density between the ground and excited state and were visualized using the VESTA package.[12] The RMSD of ground state and excited singlet state was visualized using VMD program.[13]

Electrochemistry measurements. Cyclic Voltammetry (CV) analysis was performed on an Electrochemical Analyzer potentiostat model 620E from CH Instruments at a sweep rate of 100

mV/s. Differential pulse voltammetry (DPV) was conducted with an increment potential of 0.004 V and a pulse amplitude, width, and period of 50 mV, 0.05, and 0.5 s, respectively. Samples were prepared in DMF solutions, which were degassed by sparging with DMF-saturated nitrogen gas for 5 minutes prior to measurements. All measurements were performed using 0.1 M DMF solution of tetra-*n*-butylammonium hexafluorophosphate, [nBu$_4$N]PF$_6$]. An Ag/Ag$^+$ electrode was used as the reference electrode while a platinum electrode and a platinum wire were used as the working electrode and counter electrode, respectively. The redox potentials are reported relative to a saturated calomel electrode (SCE) with a ferrocenium/ferrocene (Fc/Fc$^+$) redox couple as the internal standard (0.45 V vs SCE).[14] The HOMO and LUMO energies were determined using the relation $E_{HOMO/LUMO} = -(E_{ox} / E_{red} + 4.8)$ eV, where E_{ox} and E_{red} are the onset of anodic and cathodic peak potentials, respectively calculated from DPV relative to Fc/Fc$^+$.[15]

Photophysical measurements. Optically dilute solutions of concentrations on the order of 10^{-5} or 10^{-6} M were prepared in spectroscopic grade solvents for absorption and emission analysis. Absorption spectra were recorded at room temperature on a Shimadzu UV-2600 double beam spectrophotometer with a 1 cm quartz cuvette. Molar absorptivity determination was verified by linear regression analysis of values obtained from at least four independent solutions at varying concentrations with absorbance ranging from 0.025 to 0.100.

For emission studies, steady-state emission, excitation spectra and time-resolved emission spectra were recorded at room temperature using an Edinburgh Instruments FS5 fluorimeter. Samples were excited at 340 nm for steady-state measurements. Photoluminescence quantum yields for solutions were determined using the optically dilute method,[16] in which four sample solutions with absorbances of ca. 0.10, 0.075, 0.050 and 0.025 at 360 nm were used. The Beer-Lambert law was found to remain linear at the concentrations of the solutions. For each sample, linearity between absorption and emission intensity was verified through linear regression analysis with the Pearson regression factor (R^2) for the linear fit of the data set surpassing 0.9. Individual relative quantum yield values were calculated for each solution and the values reported represent the slope obtained from the linear fit of these results. The quantum yield of the sample, F_{PL}, can be determined by the equation $\Phi_{PL} = (\Phi_r * \frac{A_r}{A_s} * \frac{I_s}{I_r} * \frac{n_s^2}{n_r^2})$,[17] where A stands for the absorbance at the excitation wavelength (λ_{exc}: 338 nm), I is the integrated area under the corrected emission curve and n is the refractive index of the solvent with the subscripts “s” and “r” representing sample and reference respectively. F_r is the absolute quantum yield of the external reference quinine sulfate (Φ_r = 54.6% in 1 *N* H_2SO_4).[18]

An integrating sphere (Edinburgh Instruments FS5, SC30 module) was employed for the photoluminescence quantum yield measurements of thin film samples. The Φ_{PL} of the films were then measured in air and in N_2 by purging the integrating sphere with N_2 gas flow for 2 min. The photophysical properties of the film samples were measured using an Edinburgh Instruments FS980 fluorimeter. Time-resolved PL measurements of the thin films were carried out using the multi-channel scaling (MCS) and time-correlated single-photon counting (TCSPC) technique. The samples were excited at 379 nm by a pulsed laser diode (PicoQuant, LDH-D-C-375, FWHM < 40 ps, pulse energy = 58.5 ± 1.2 pJ, peak power = 1.5 ± 0.3 W, laser spot diameter = 0.4 ± 0.1 mm, power density = 11.6 ± 3.7 mW/cm^2) and were kept in a vacuum of < 8 × 10^{-4} mbar. The singlet and triplet state energies in toluene were determined from the onset values of the steady-state PL and phosphorescence spectra at 77 K. The singlet-triplet energy gap (ΔE_{ST}) was estimated from the difference in energy of the steady-state PL and phosphorescence spectra. The samples were excited by a xenon flashlamp emitting at 340 nm (EI FS5, SC-70). Phosphorescence spectra of toluene glass were measured with a time-gated window of 1-10 ms. The ΔE_{ST} of in film was estimated from the onset of the SS spectrum and phosphorescence spectrum at 77 K. The film was excited by a flash lamp emitting at 340 nm (EI FS5). Phosphorescence spectra of doped film were measured with time-gated window of 1-10 ms.

Fitting of time-resolved luminescence measurements: Time-resolved PL measurements were fitted to a sum of exponentials decay model, with chi-squared (χ^2) values between 1 and 2, using the EI FLS980 Each component of the decay is assigned with a weight, (w_i), which is the contribution of the emission from each component to the total emission.

The average lifetime was then calculated using the following expressions:

1. Two exponential decay model:

$$\tau_{AVG} = \tau_1 w_1 + \tau_2 w_2 \qquad \text{(S1)}$$

with weights defined as $w_1 = \frac{A1\tau_1}{A1\tau_1 + A2\tau_2}$ and $w_2 = \frac{A2\tau_2}{A1\tau_1 + A2\tau_2}$ where A_1 and A_2 are the preexponential-factors of each component.

2. Three exponential decay model:

$$\tau_{AVG} = \tau_1 w_1 + \tau_2 w_2 + \tau_3 w_3 \qquad \text{(S2)}$$

with weights defined as $w_1 = \frac{A1\tau_1}{A1\tau_1 + A2\tau_2 + A3\tau_3}$, $w_2 = \frac{A2\tau_2}{A1\tau_1 + A2\tau_2 + A3\tau_3}$ and $w_3 =$

$\frac{A3\tau_3}{A1\tau_1 + A2\tau_2 + A3\tau_3}$ where A_1, A_2 and A_3 are the preexponential-factors of each component.

OLED Fabrication and Characterization: The OLED devices were fabricated in a bottom-emitting structure via thermal evaporation in a high vacuum at a base pressure of $<5\times10^{-7}$ mbar. A pre-patterned glass substrate coated with indium doped tin oxide (ITO) was cleaned sequentially by ultrasonication in acetone, and isopropanol for 15 minutes. The temperature of ultrasonication bath was set at 60-70 ^{o}C. The cleaned substrate was exposed to oxygen plasma for 3 min to remove all dust and organics on the ITO surface and to increase the work function of ITO anode for better hole injection from the anode to organic layer. The substrate was loaded in the thermal evaporator. Organic layers were deposited at a rate of 0.3-1.0 Å/s, monitored using a quartz crystal. The electron injection layer, LiF, was deposited at a rate of 0.05 Å/s, while the Al cathode was deposited initially with a rate of 0.5 Å/s to obtain 10 nm thickness and after that the rate of Al cathode was increased to 3 Å/s. Two custom-made shadow masks were used to define the area of the evaporations. The organic layers and LiF were evaporated with a same shadow mask, but Al were evaporated with the other mask. The active area of the OLED was 2 mm^2, determined by the spatial overlap of the anode and cathode electrodes. All the devices were encapsulated with glass lids and UV epoxy resin inside a N_2 filled globe box. The luminance-current-voltage characteristics were measured in an ambient environment using a Keithley 2400 source meter and a homemade photodiode circuit connected to a Keithley 2000 multimeter for the voltage reading. The external quantum efficiency was calculated assuming Lambertian emission pattern for the OLEDs. The electroluminescence spectra were recorded by an Andor DV420-BV CCD spectrometer.

Experimental Section

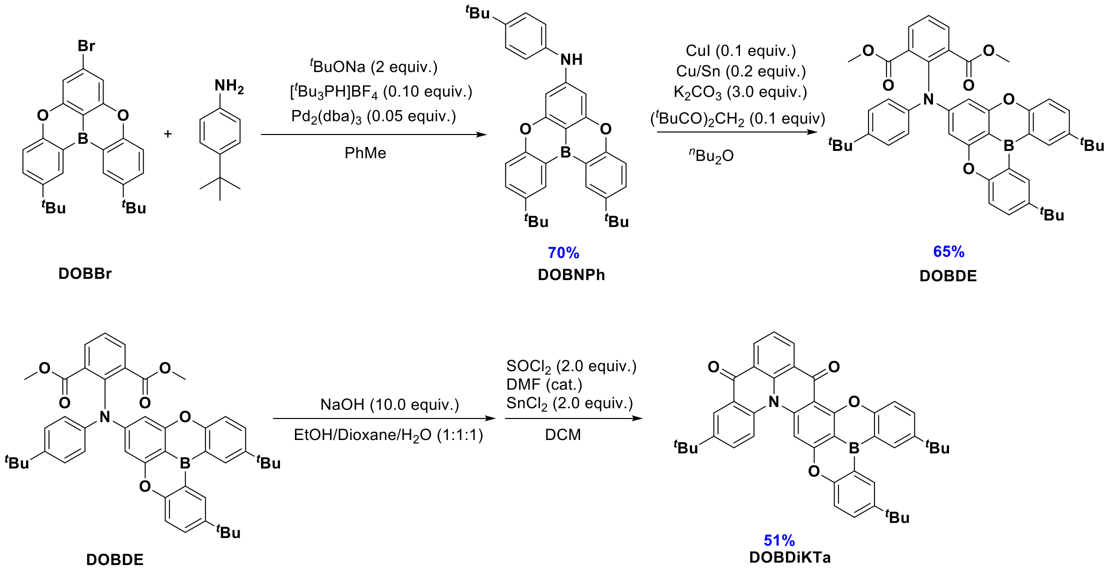

Scheme **S1**. Synthetic route of **DOBDiKTA**.

2,12-di-*tert*-butyl-N-(4-(*tert*-butyl)phenyl)-5,9-dioxa-13b-boranaphtho[3,2,1-de]anthracen-7-amine (**DOBNPh**)

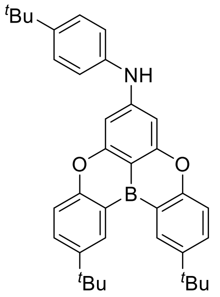

DOBBr (4.00 g, 8.67 mmol, 1.0 equiv.), 4-(*tert*-butyl) aniline (2.59 g, 17.35 mmol, 2.0 equiv.), Sodium *tert*-butoxide (1.67 g, 17.35 mmol, 2.0 equiv.), $Pd_2(dba)_3$ (0.25 g, 0.87 mmol, 0.1 equiv.) and were added to a Schlenk flask containing 20 mL of anhydrous toluene. After degassing the flask, the reaction system was placed under a nitrogen atmosphere. The mixture was heated at 115 °C for 24 h. After cooling to room temperature, DCM (50 mL) was added to the mixture. The mixture was washed with a saturated NaCl aqueous solution (50 mL × 2). The collected organic phase was dried over anhydrous sodium sulfate and concentrated under reduced pressure. The collected crude product was purified by silica gel column chromatography (EtOAc: hexane = 1:10, **R$_f$**: 0.30) to afford the target compounds with white powder. **Yield**. 3.2 g, 70%. **Mp**: 271-373 °C. **^{1}H NMR (400 MHz, CD_2Cl_2)** δ 8.75 (d, J = 2.5 Hz, 2H), 7.77 (dd, J = 8.8, 2.5 Hz, 2H), 7.49 – 7.41 (m, 4H), 7.30 – 7.25 (m, 2H), 6.77 (s, 2H), 6.30 (s, 1H), 1.52 (s, 18H), 1.40 (s, 9H). **^{13}C NMR (101 MHz, CD_2Cl_2)** δ 159.15, 158.56, 151.17, 146.83, 144.92, 137.91, 130.74, 130.13, 126.34, 121.37, 117.46, 94.50, 34.41, 34.30, 31.32, 31.19. **HRMS [M+H$^+$]**: Calculated: 530.3225 ($C_{36}H_{40}BNO_4$); Found: 530.3239.

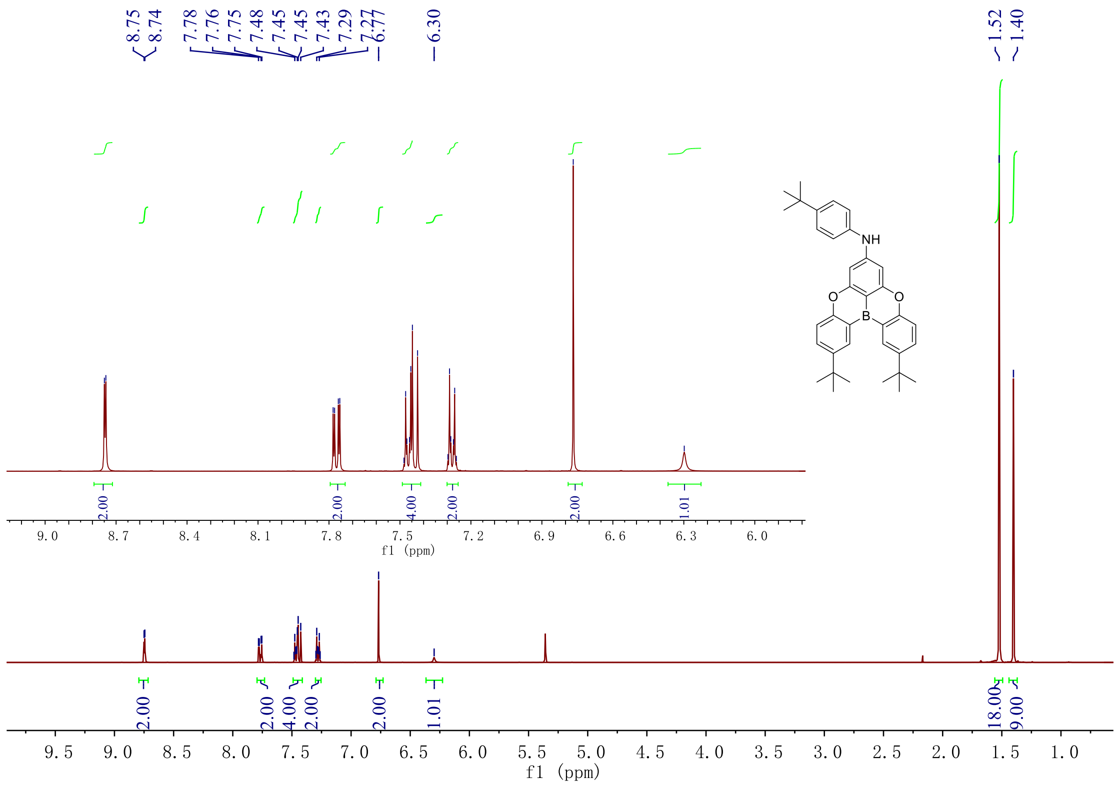

Figure **S1**. ^{1}H NMR spectrum of **DOBNPh** in CD_2Cl_2.

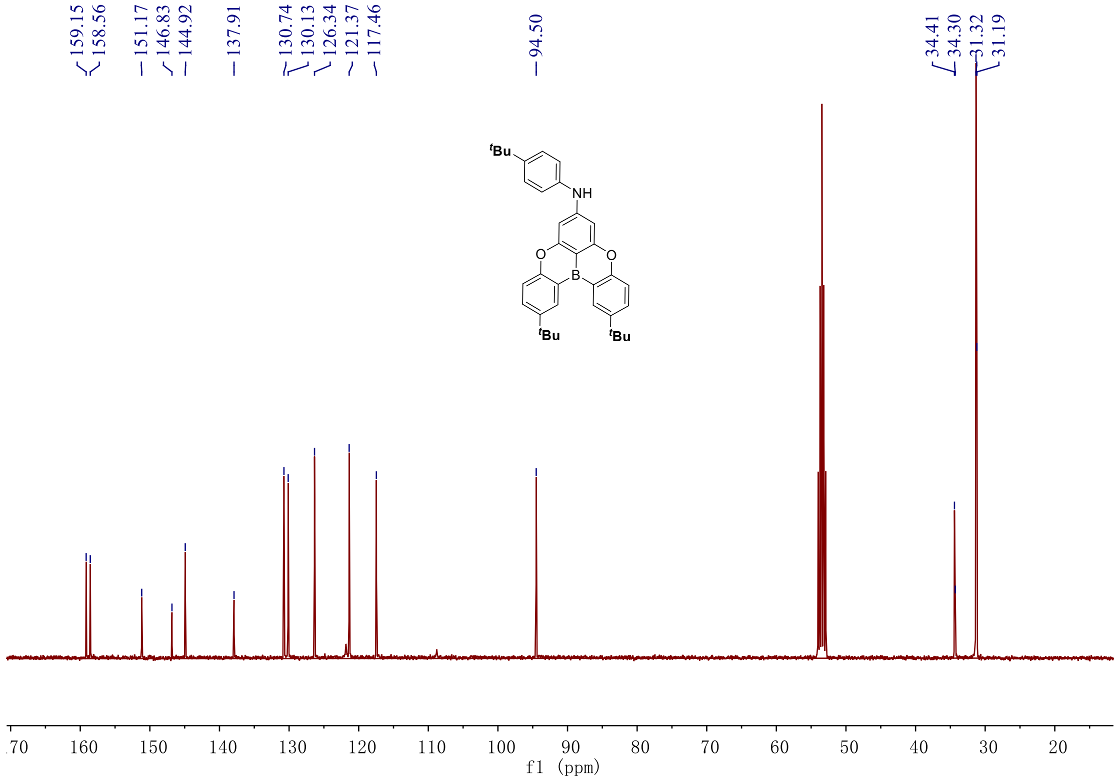

Figure **S2**. ^{13}C NMR spectrum of **DOBNPh** in CD_2Cl_2.

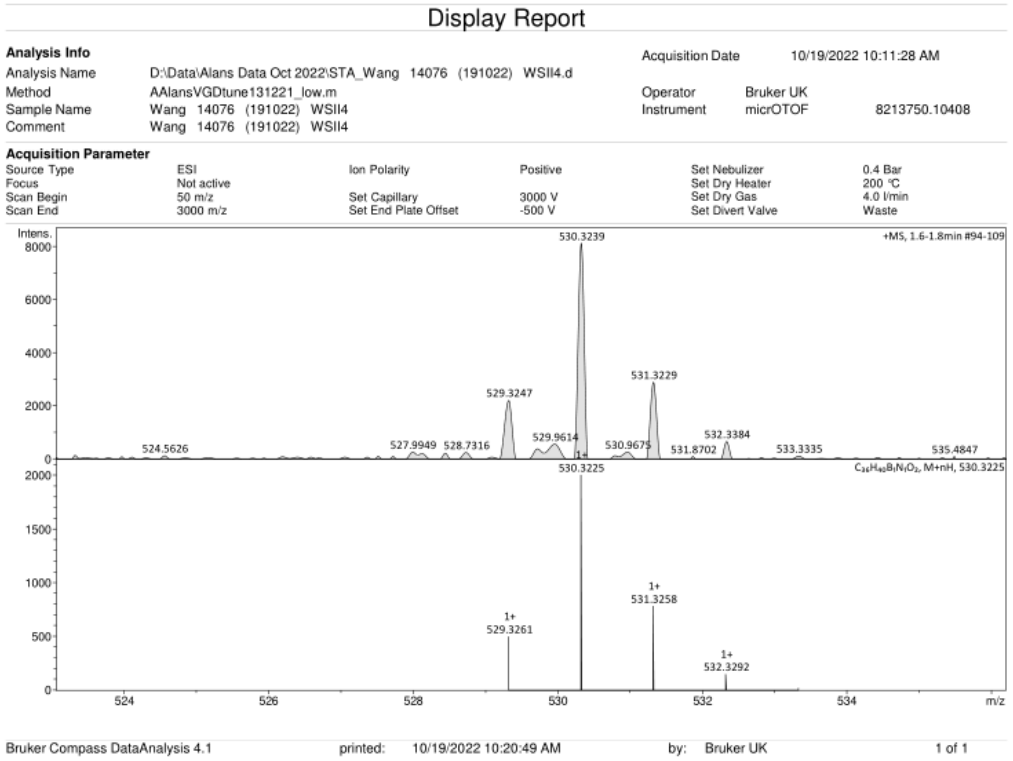

Figure **S3**. HRMS spectrum of **DOBNPh.**

dimethyl 2-((4-(*tert*-butyl)phenyl)(2,12-di-*tert*-butyl-5,9-dioxa-13b-boranaphtho[3,2,1-de] anthracen -7-yl)amino)isophthalate (**DOBDE**)

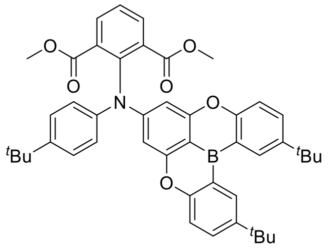

DOBNPh (3.00 g, 5.67 mmol, 1.0 equiv.), 2-bromoisophthalic acid dimethyl ester (3.09 g, 11.33 mmol, 2.0 equiv.), potassium carbonate (2.35 g, 17.00 mmol, 3.0 equiv.), CuI (0.11 g, 0.57 mmol, 0.1 equiv.), Cu/Sn (0.21 g, 1.13 mmol, 0.2 equiv.) and 2,2,6,6-Tetramethyl-3,5-heptanedione ($(^{t}BuCO)_2CH_2$, 0.11 g, 0.57 mmol, 0.1 equiv.) were added to a Schlenk flask containing 30 mL of anhydrous dibutyl ether. After degassing the flask, the reaction system was placed under a nitrogen atmosphere. The mixture was heated at 145 °C for 3 days. After cooling to room temperature, DCM (50 mL) was added to the mixture. The mixture was washed with a saturated NaCl aqueous solution (50 mL × 3). The collected organic phase was dried over anhydrous sodium sulfate and concentrated under reduced pressure. The collected crude product was purified by silica gel column chromatography (EtOAc : hexane = 1:4, $\mathbf{R}_f$: 0.35) to afford the target compounds with yellow powder. **Yield**. 2.7 g, 65%. **Mp**: 239-241 °C. **^{1}H NMR (400 MHz, CD_2Cl_2)** δ 8.75 (d, J = 2.4 Hz, 2H), 7.92 (d, J = 7.8 Hz, 2H), 7.76 (dd, J = 8.8, 2.4 Hz, 2H), 7.51 (s, 1H), 7.39 (dd, J = 8.7, 4.3 Hz, 4H), 7.15 (d, J = 8.6 Hz, 2H), 6.66 (s, 2H), 3.51 (s, 6H), 1.51 (s, 18H), 1.38 (s,

9H). **^{13}C NMR (101 MHz, CD_2Cl_2)** δ 166.73, 158.63, 158.38, 153.65, 147.34, 144.98, 143.46, 142.61, 133.95, 132.94, 130.91, 130.13, 126.55, 125.75, 124.54, 121.80, 117.50, 99.20, 34.41, 34.33, 31.29, 31.11, 22.67, 13.90. **HRMS [M+Na$^+$]**: Calculated: 744.3467 ($C_{46}H_{48}BNO_6$); Found:744.3501.

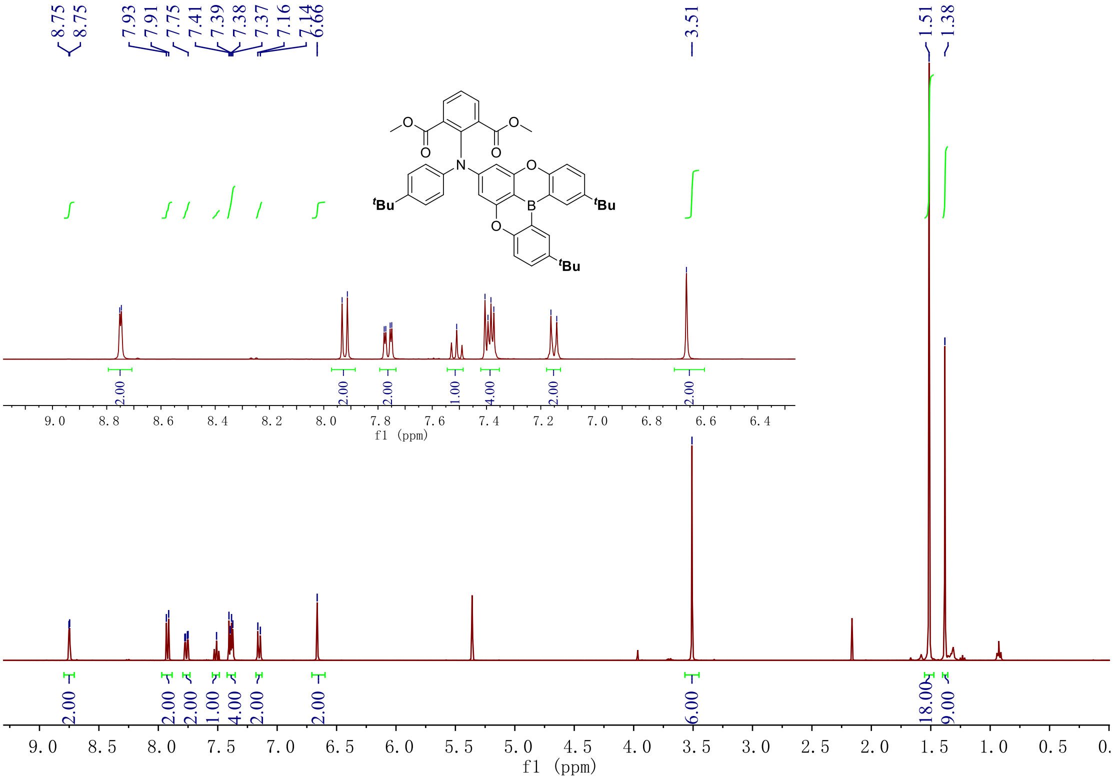

Figure **S4**. ^{1}H NMR spectrum of **DOBDE** in CD_2Cl_2.

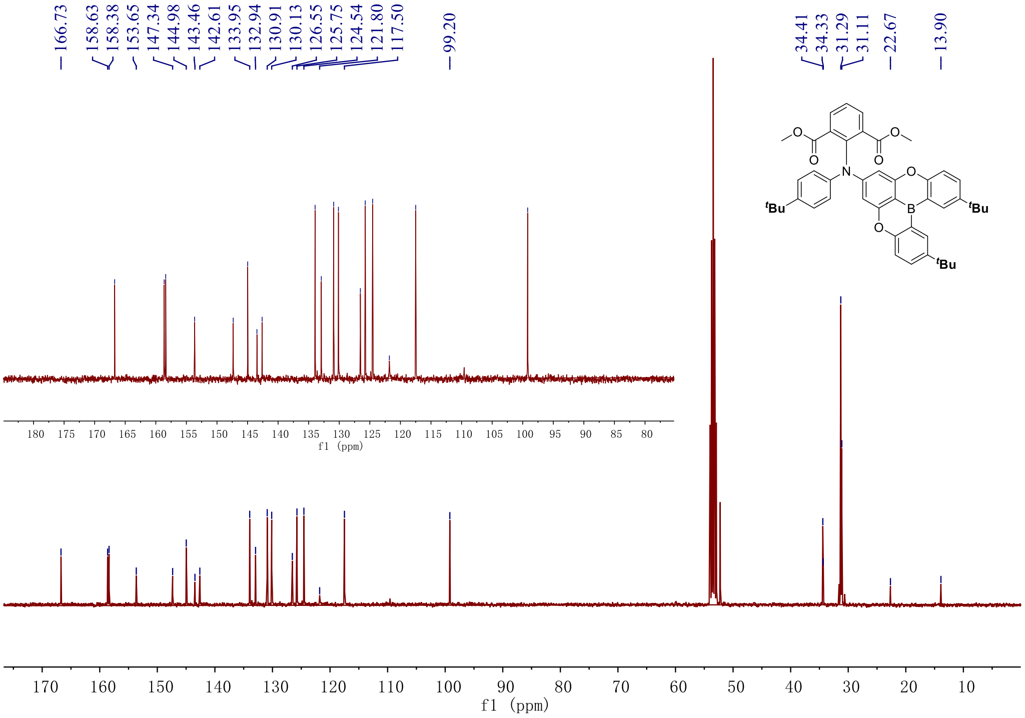

Figure **S5**. ^{13}C NMR spectrum of **DOBDE** in CD_2Cl_2.

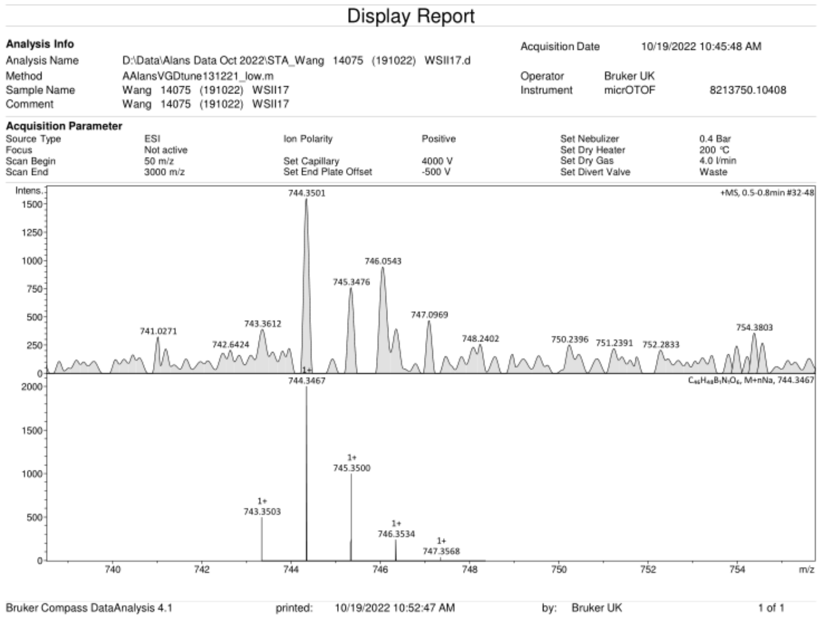

Figure **S6.** HRMS spectrum of **DOBDE.**

3,6,13-tri-*tert*-butyl-9,20-dioxa-10b-aza-4b-boradinaphtho[3,2,1-de:1',2',3'-qr]pentacene-15,19-dione (**DOBDiKTa**)

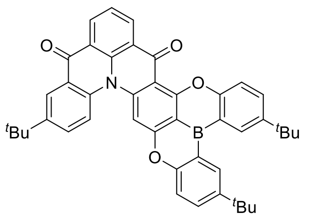

DOBDE (2.5 g, 3.46 mmol, 1 equiv.) was combined with sodium hydroxide (1.40 g, 34.60 mmol, 10 equiv.) in 20 mL of an ethanol/water (1:1) mixture. The reaction was heated to reflux for 12 h. After cooling to room temperature, the pH was adjusted to 2-3 by addition of dilute hydrochloric acid. The diacid precipitated as a light green solid and was collected by vacuum filtration, washed thoroughly with water, dried under vacuum (2.20, 92% yield) and used without further purification and characterization. The diacid (0.60 g, 0.87 mmol, 1 equiv.) was dispersed in 20 mL dichloromethane under a nitrogen atmosphere. To the reaction mixture were added sequentially thionyl chloride (0.16 mL, 2.16 mmol, 2.5 equiv.) and 1 drop of DMF. After 3 h under reflux, the reaction mixture was cooled to room temperature. Under a positive flow of nitrogen, $SnCl_2$ (0.82 g, 4.33 mmol, 5 equiv.) was added slowly. After refluxing for 12 h, the reaction mixture was cooled to room temperature and the reaction quenched by dropwise addition of water. The mixture was combined with dichloromethane (50 mL). The mixture was washed with a saturated NaCl aqueous solution (50 mL×3). The organic fractions were combined, and the solvent volume was concentrated under reduced pressure. The collected crude product was purified by silica gel column chromatography (DCM: hexane = 2:1, $\mathbf{R}_f$: 0.5) to afford the target compound as a

yellow powder. **Yield**. 0.4 g, 51%. **Mp**: 354-355 °C. **^{1}H NMR (400 MHz, $CDCl_3$)** δ 8.78 – 8.71 (m, 3H), 8.67 (dd, J = 7.7, 1.7 Hz, 1H), 8.51 (d, J = 2.4 Hz, 1H), 8.26 (d, J = 8.9 Hz, 1H), 7.86 – 7.80 (m, 2H), 7.78 (dd, J = 8.8, 2.5 Hz, 1H), 7.74 (d, J = 8.8 Hz, 1H), 7.67 – 7.62 (m, 2H), 7.42 (d, J = 8.7 Hz, 1H), 1.53 – 1.48 (m, 27H). **^{13}C NMR (101 MHz, $CDCl_3$)** δ 179.02 (s), 176.70, 159.79, 158.61, 158.33, 157.96, 148.85, 146.24, 146.09, 146.02, 138.65, 137.79, 132.04, 131.84, 131.73, 131.56, 130.60, 130.51, 129.60, 126.40, 125.34, 123.73, 123.67, 122.97, 121.50, 120.69, 119.07, 117.66, 109.73, 100.08, 34.95, 34.68, 34.64, 31.54, 31.30. **Anal. Calcd. For $C_{44}H_{40}BNO_4$**: C 80.36%, H 6.13%, N 2.13% **Found**: C 79.73%, H 6.13%, N 2.23%. **HRMS [M^{+}]**: Calculated: 658.3123 ($C_{44}H_{40}BNO_4$); Found: 658.3121. 99.89% pure on HPLC analysis, retention time 10.833 minutes in 100% THF.

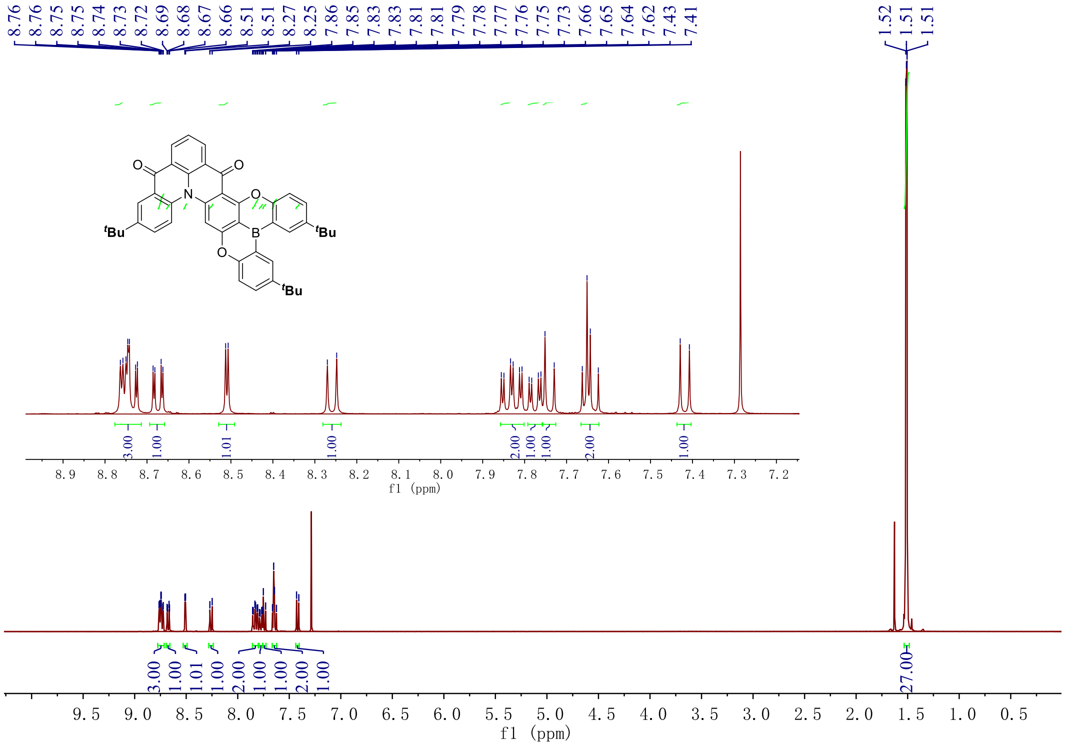

Figure **S7**. ^{1}H NMR spectrum of **DOBDiKTa** in $CDCl_3$.

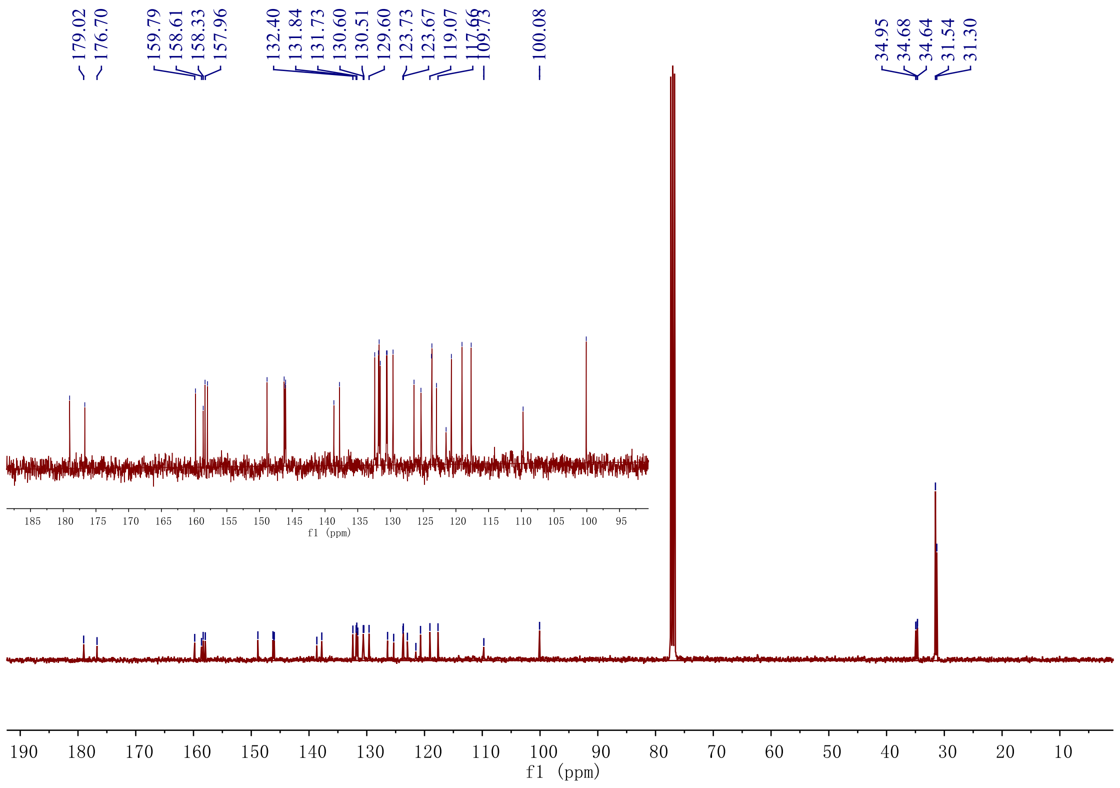

Figure **S8**. ^{13}C NMR spectrum of **DOBDiKTa** in $CDCl_3$.

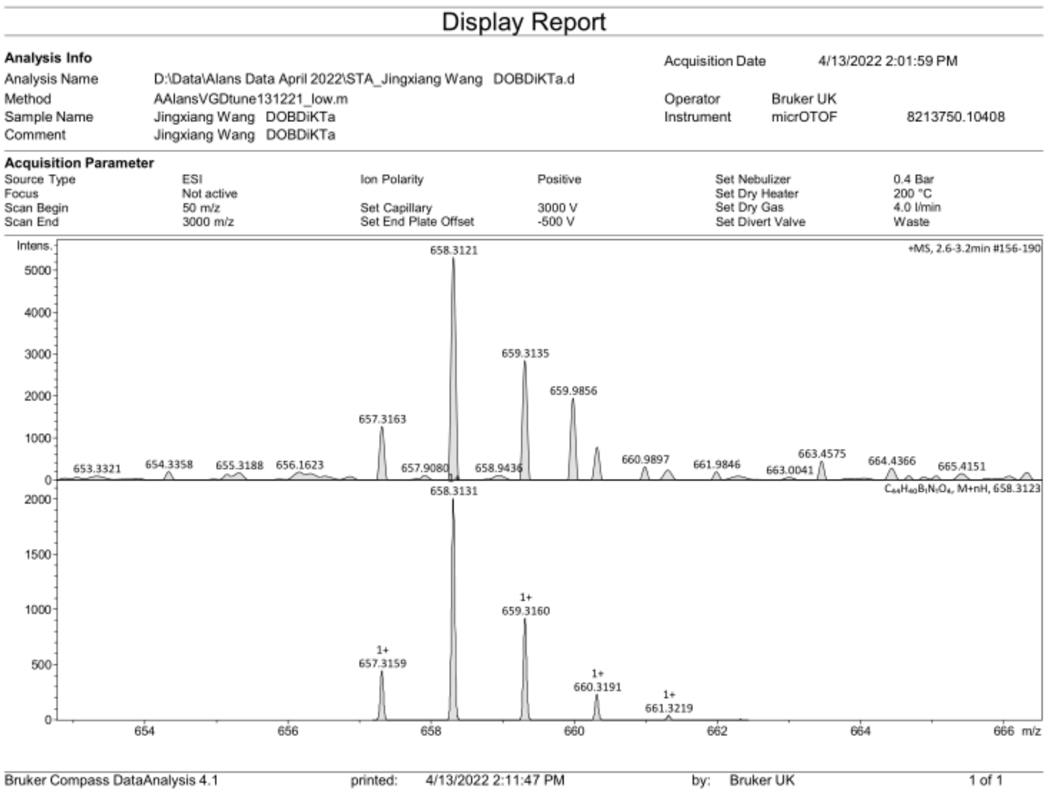

Figure **S9**. HRMS spectrum of **DOBDiKTa**.

NOTE: Please submit ca. 10 mg of sample

**Sample reference number**	WS-II21
**Name of Compound**	DIKTADOB
**Molecular formula**	C44H40BNO4
**Stability**	
**Hazards**	
**Other Remarks**	

Analysis type:

Single ☐ **Duplicate** ☑ **Triplicate** ☐

Analysis Result:

Element	Expected %	Found (1)	Found (2)	Found (3)
Carbon	80.36	79.38	79.73	
Hydrogen	6.13	6.05	6.13	
Nitrogen	2.13	2.23	2.23	
Oxygen				

Authorising Signature:

**Date completed**	23.05.22
**Signature**	[illegible]
**comments**	

Figure **S10**. Elemental analysis data of **DOBDiKTa**.

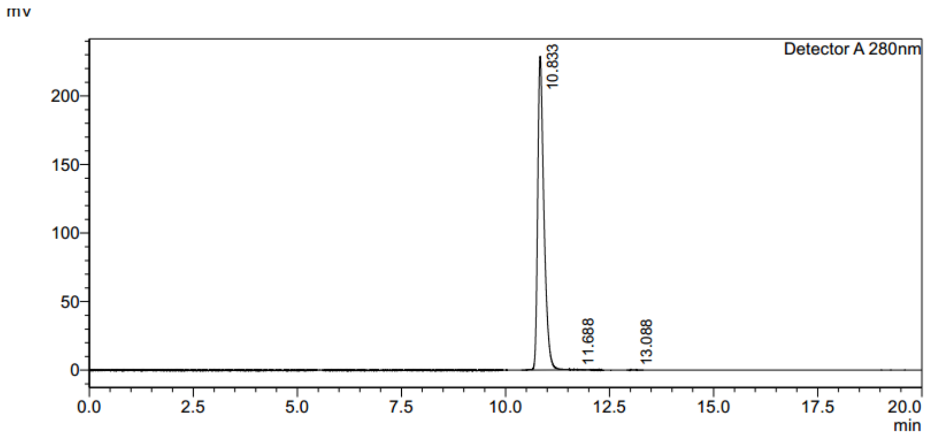

<Peak Table>

Detector A 280nm

Peak#	Ret. Time	Area	Height	Area%	Area/Height	Width at 5% Height
1	10.833	2404941	228183	99.891	10.540	0.371
2	11.688	1320	126	0.055	10.471	0.265
3	13.088	1308	126	0.054	10.394	0.289
Total		2407569	228435	100.000		

Figure **S11**. HPLC spectrum of **DOBDiKTa**.

Computations

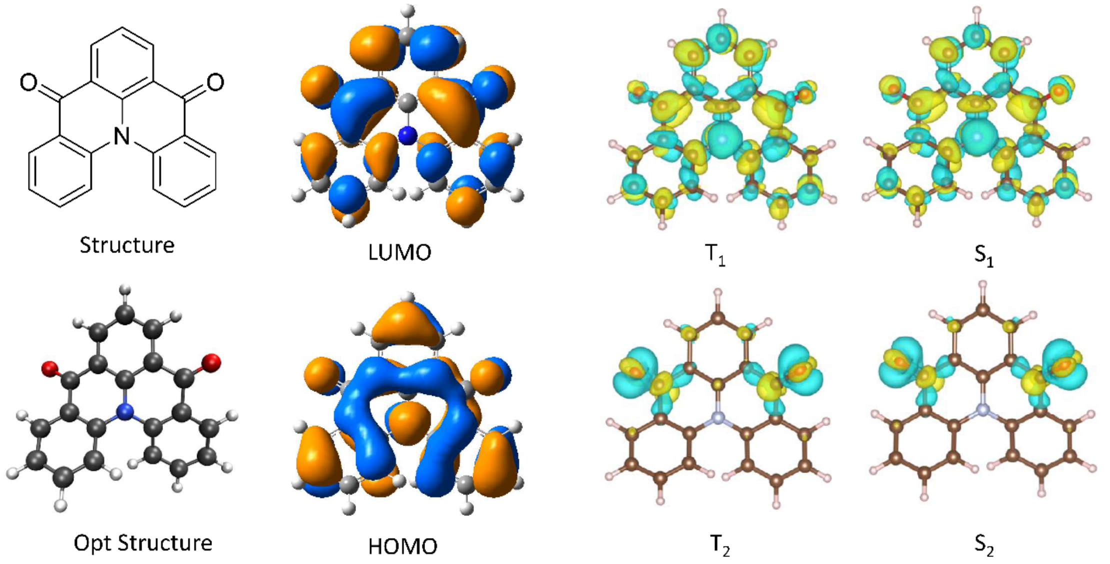

Figure S12. Chemical structure, optimized structure of **DiKTa** and HOMO and LUMO orbitals calculated in the gas phase at the PBE0/6-31G(d,p) level and difference density plots of S_1, S_2, T_1 and T_2 excited states calculated in the gas phase at the SCS-ADC(2)/cc-pVDZ level.

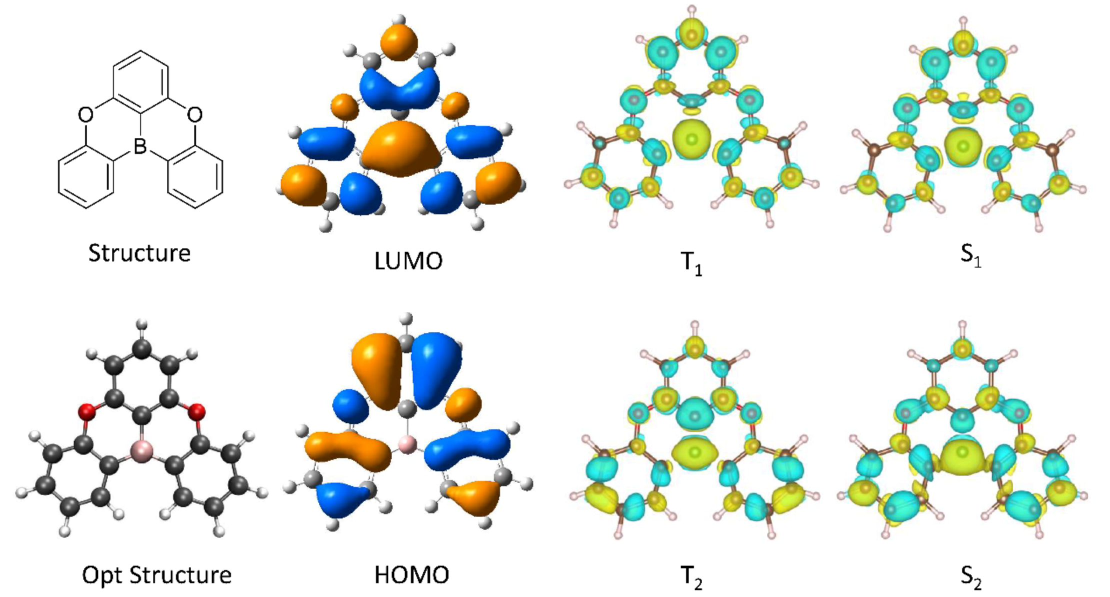

Figure S13. Chemical structure, optimized structure of **DOBNA** and HOMO and LUMO orbitals calculated in the gas phase at the PBE0/6-31G(d,p) level and difference density plots of S_1, S_2, T_1 and T_2 excited states calculated in the gas phase at the SCS-ADC(2)/cc-pVDZ level.

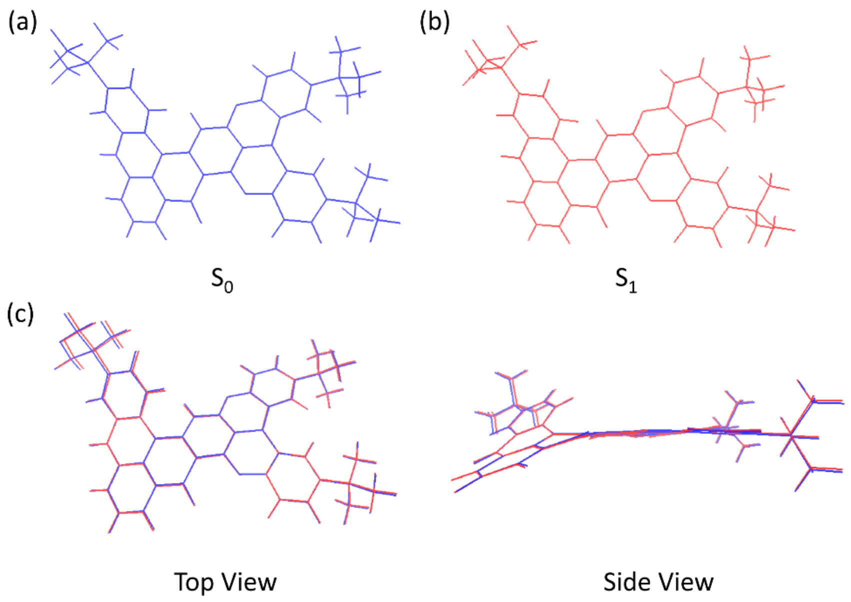

Figure **S14**. Optimized structures of the (a) ground state (S_0) and (b) excited singlet state (S_1); (c) (d) the geometric difference between the S_0 and S_1 states. The root mean square deviation (RMSD) value between the two configurations is 0.10155.

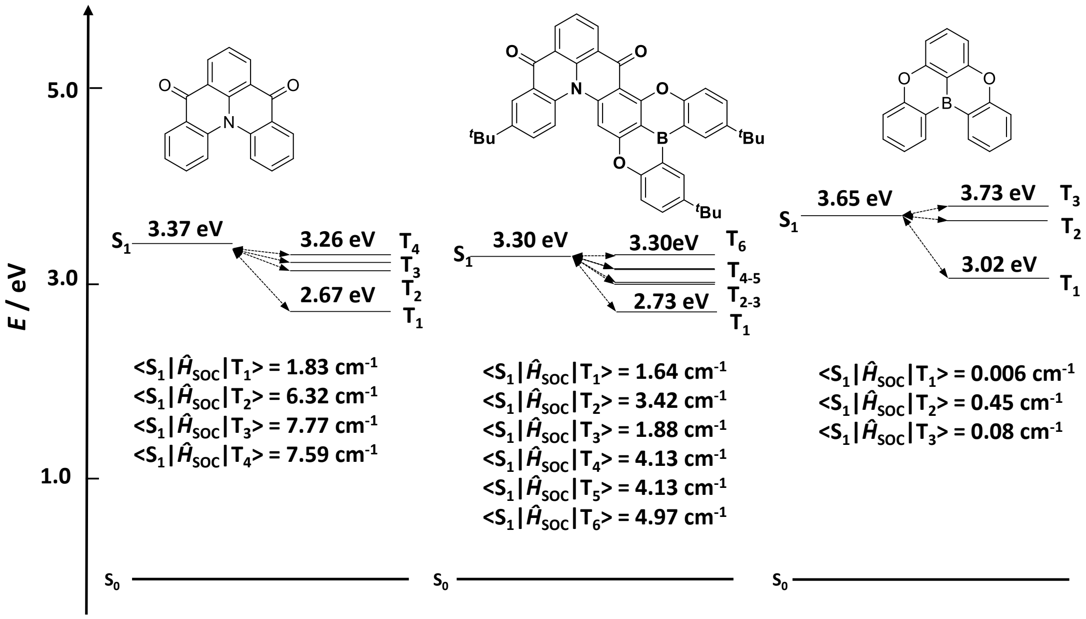

Figure **S15**. Spin-orbit coupling matrix element (SOCME) for DiKTa, DOBDiKTa and DOBNA emitters.

Transition	NTOs	FMOs	
$S_0 \longrightarrow S_1$<br>HOMO → LUMO 90%<br>HOMO-3 → LUMO 3%<br>*f* =0.41	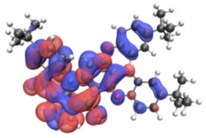	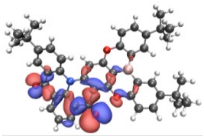<br>HOMO-3	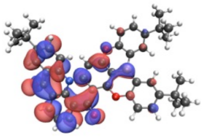<br>LUMO
$S_0 \longrightarrow S_6$<br>HOMO-1 → LUMO+1 81%<br>HOMO-1 → LUMO 6%<br>HOMO → LUMO+2 4%<br>*f* =0.31	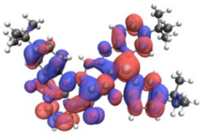	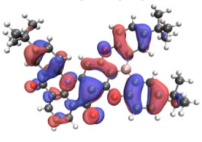<br>HOMO-1	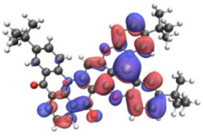<br>LUMO+1
$S_0 \longrightarrow S_8$<br>HOMO → LUMO+2 82%<br>HOMO-1 → LUMO+1 4%<br>*f* =0.12	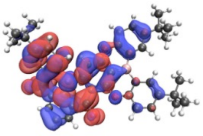	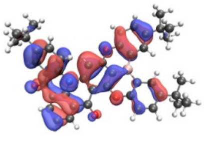<br>HOMO	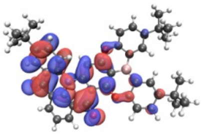<br>LUMO+2

Figure **S16**. The contribution of the molecular orbitals (MOs) of the transition from ground (S_0) to excited singlet (S_1, S_6 and S_8); the natural transition orbitals (NTOs) and the related front molecular orbitals (FMOs).

Photophysical characterization

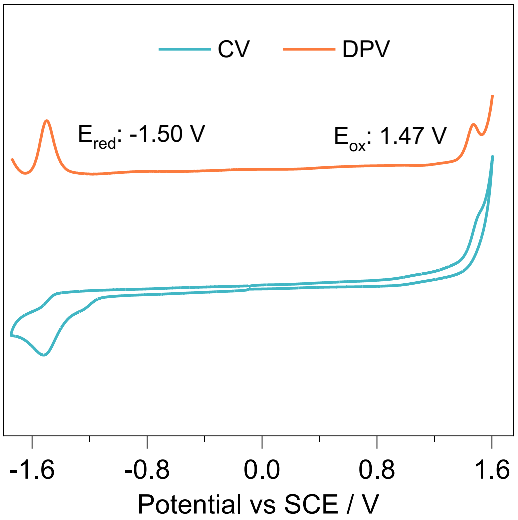

Figure **S17**. Cyclic voltammogram (CV) and differential pulse voltammetry (DPV) in degassed DMF with 0.1 M [^{n}Bu$_4$N]PF$_6$ as the supporting electrolyte and Fc/Fc$^+$ as the internal reference versus SCE (0.45 V vs. SCE).[14]

Table **S1.** Electrochemical data

Material	E_{ox} /V[a]	E_{red} /V[a]	HOMO / eV[b]	LUMO /eV[b]
**DOBDiKTa**	1.47	-1.50	-5.78	-2.80

[a] E_{ox} and E_{red} are the peak of anodic and cathodic potentials from DPV versus SCE. In degassed DMF with 0.1 M [^{n}Bu$_4$N]PF$_6$ as the supporting electrolyte and Fc/Fc$^+$ as the internal reference (0.45 V vs. SCE).[14] [b] $E_{HOMO/LUMO} = -(E_{ox} / E_{red}$ vs. Fc/Fc$^+$ + 4.8) eV.[15]

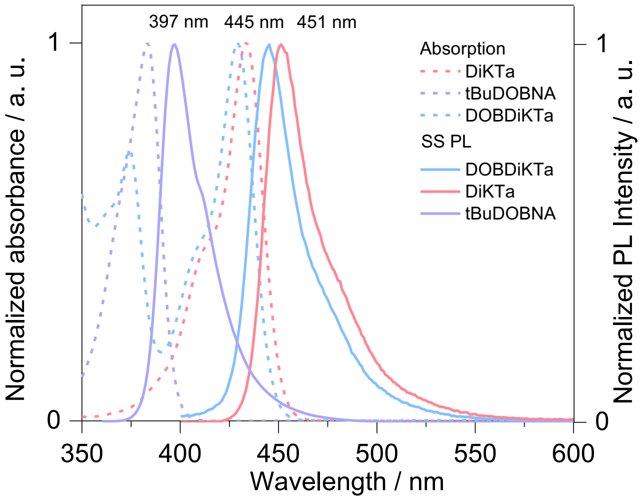

Figure **S18**. Absorption and steady-state PL spectra (SS) of **DiKTa**, **tBuDOBNA** and **DOBDiKTa** in toluene at room temperature (λ_{exc} = 340 nm).

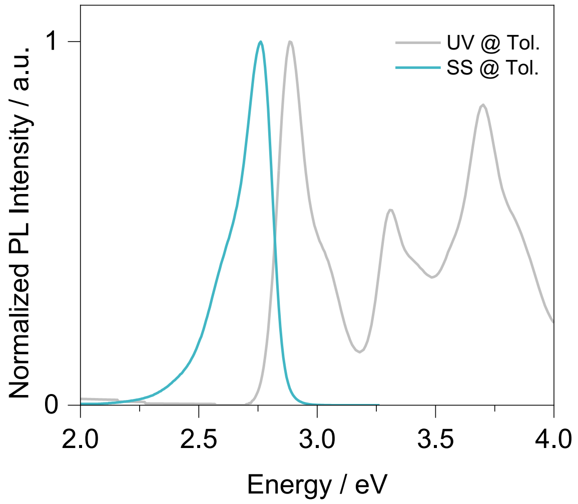

Figure **S19**. Absorption and steady-state PL spectra (SS) in toluene at room temperature (λ_{exc} = 340 nm) after Jacobian transformation.[19]

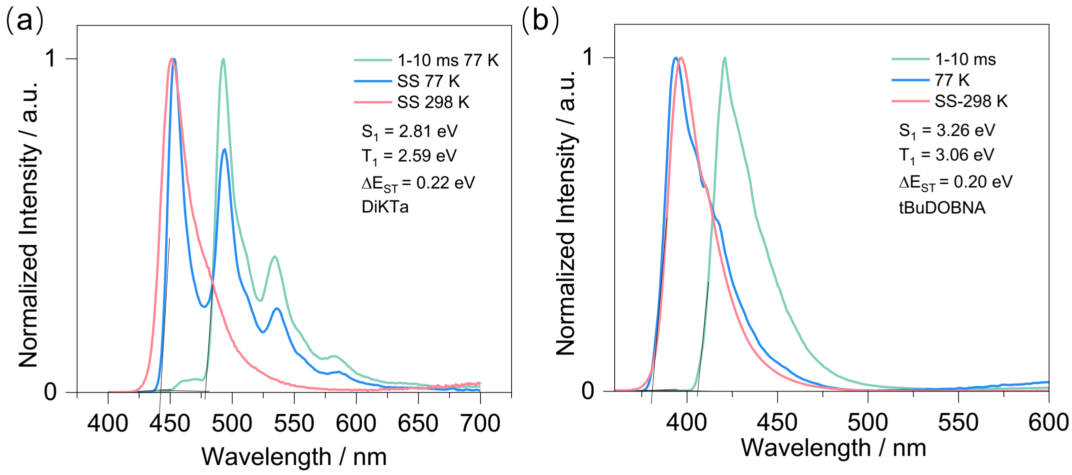

Figure **S20**. Prompt PL and delayed emission spectra of (a) **DiKTa** and (b) **tBuDOBNa** measured in dilute toluene solution at 77 K (λ_{exc} = 340 nm).

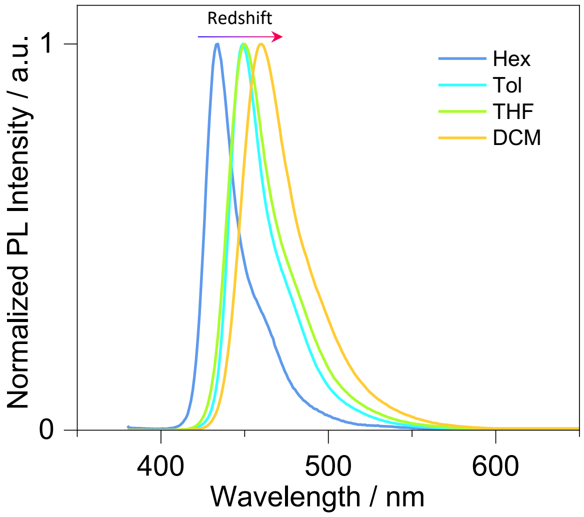

Figure **S21**. Solvatochromism PL study for **DOBDiKTa**. (λ_{exc} = 340 nm)

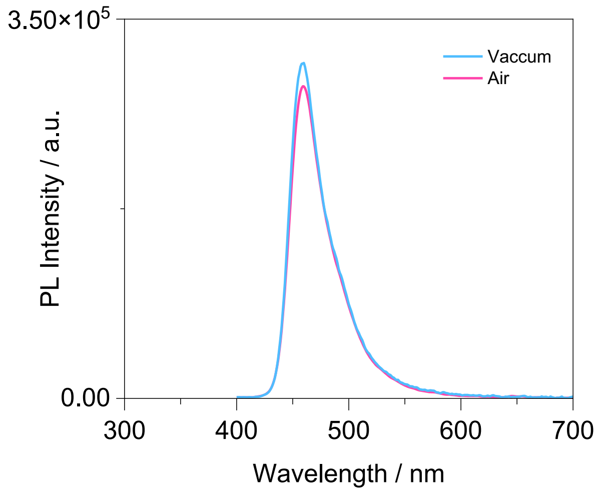

Figure **S22**. Comparison of the intensity of the PL spectra under vacuum and air (λ_{exc} = 340 nm) in 1.5 wt% doped films in mCP.

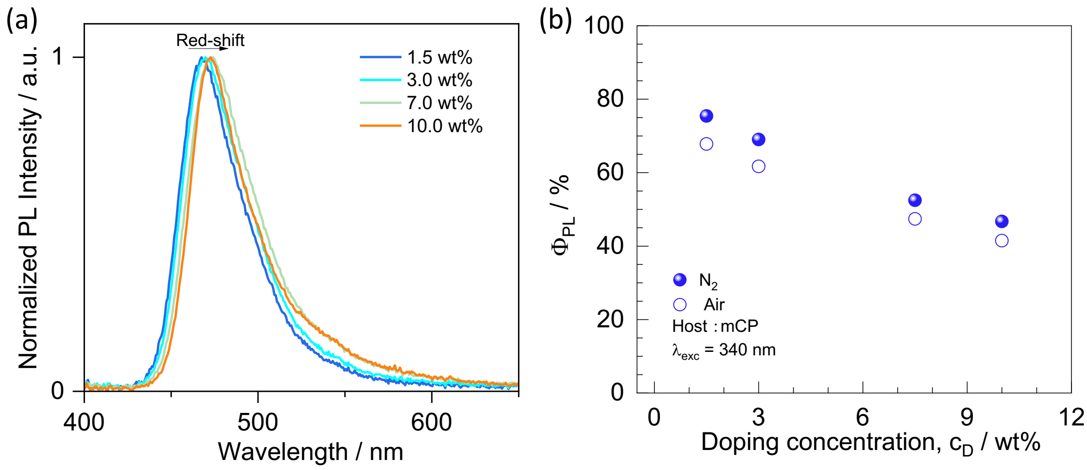

Figure **S23**. (a) Concentration-dependent PL of **DOBDiKTa** in mCP doped films under air; (b) Concentration-dependent Φ_{PL} of **DOBDiKTa** in mCP doped films under air and nitrogen. (λ_{exc}=340 nm).

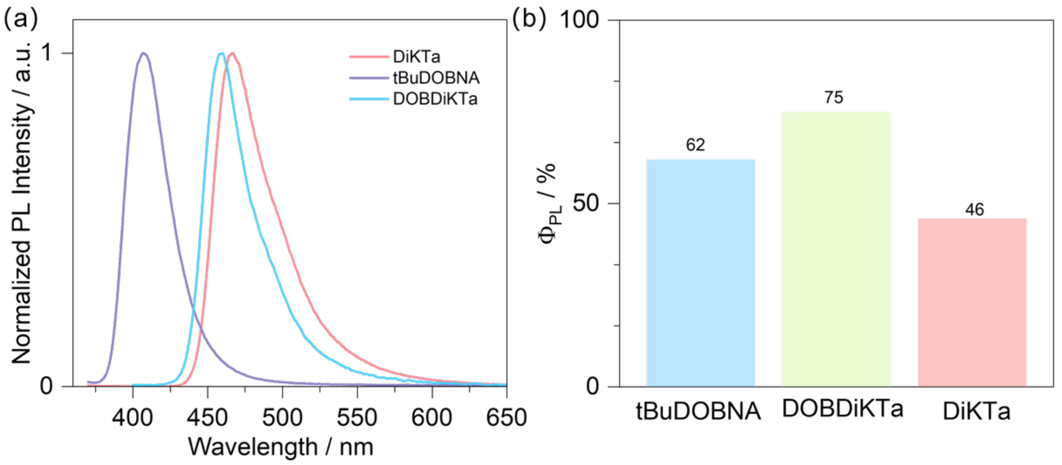

Figure **S24**. (a) SSPL and (b) Φ_{PL} of **DiKTa**, **tBuDOBNA** and **DOBDiKTa** under nitrogen as 1.5 wt% doped film in mCP (λ_{exc}=340 nm).

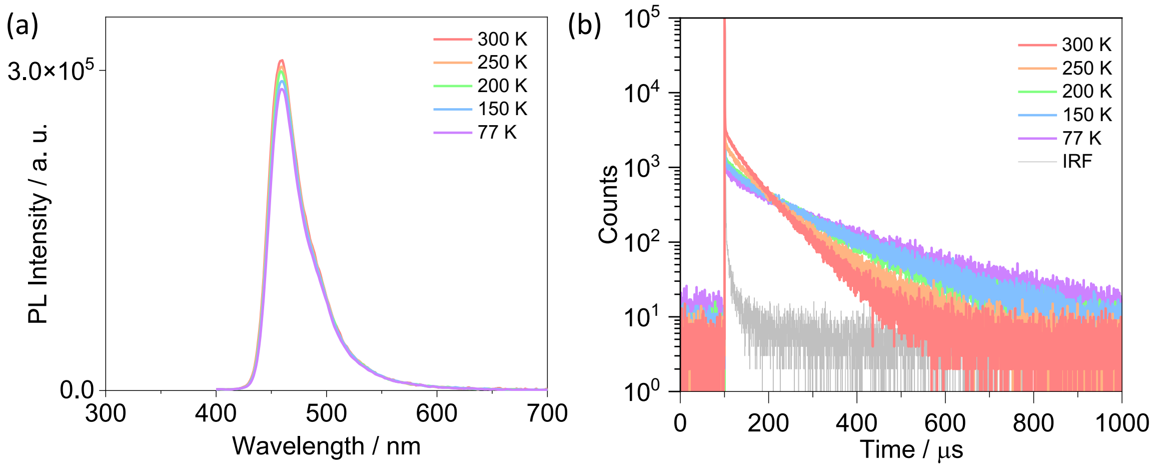

Figure **S25**. Temperature-dependent (a) SSPL spectra and (b) TRPL decay of **DOBDiKTa** in 1.5 wt% doped films in mCP, λ_{exc} = 379 nm.

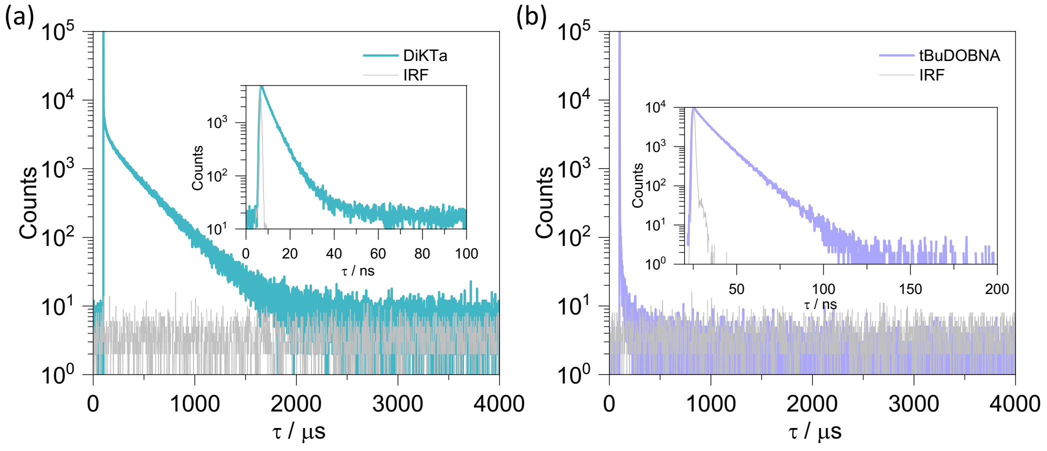

Figure **S26**. TRPL decays (λ_{exc} = 379 nm) of **DiKTa** and **tBuDOBNA** in 1.5 wt% doped films in mCP (inset figure is the PL decay of the prompt component).

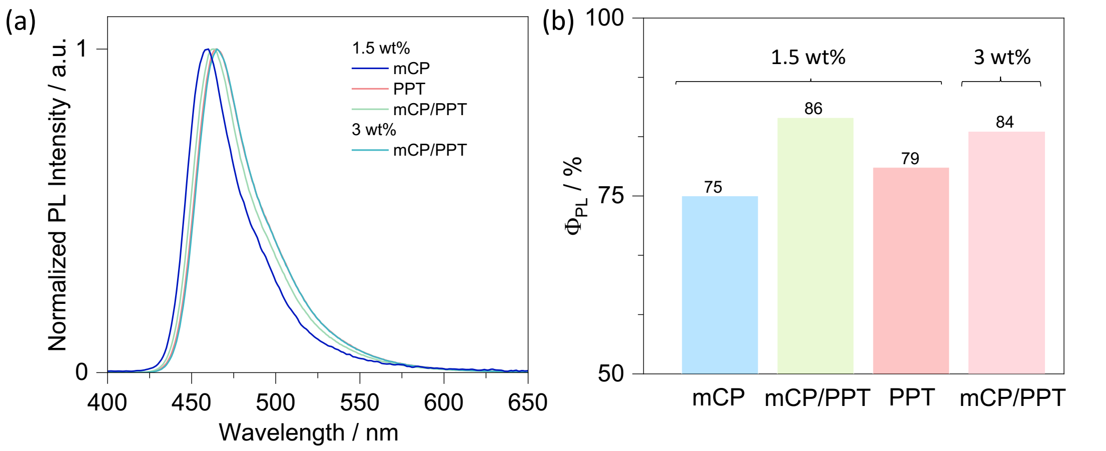

Figure **S27.** (a) SSPL in different host systems of **DOBDiKTa**; (b) Φ_{PL} of **DOBDiKTa** in different host systems under nitrogen. (λ_{exc}=340 nm).

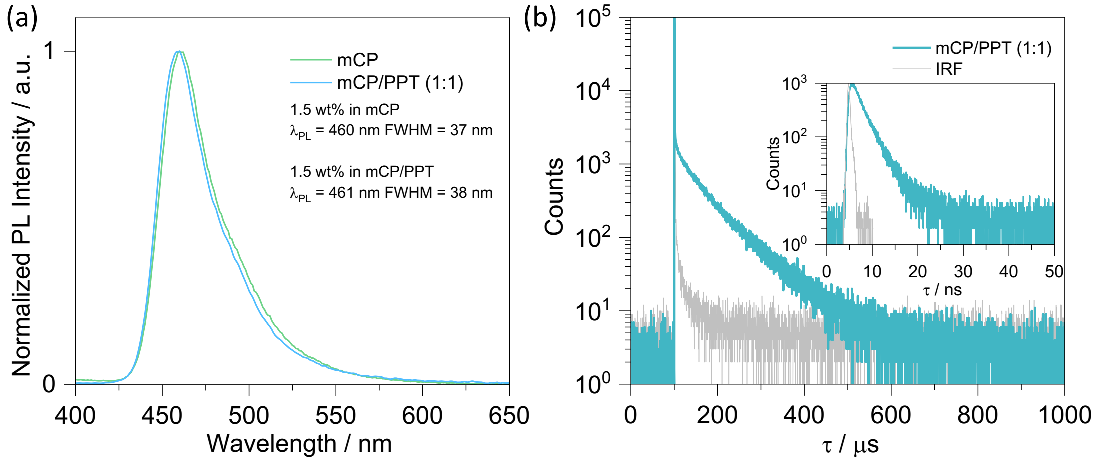

Figure **S28.** (a) Comparison of the intensity of the PL spectra in 1.5 wt% doped films in mCP and 1:1 mCP:PPT (λ_{exc} = 340 nm); and (b) Time-resolved PL decays (λ_{exc} = 379 nm) in 1.5 wt% doped films in 1:1 mCP:PPT (inset figure is the PL decay of the prompt component).

For a TADF system, the main exciton loss channels are either singlet or triplet nonradiative transition processes. Considering the PLQY of 74% for **DOB-DiKTa**, the singlet nonradiative transition process (k_{nr}^{S}) can was ignore, therefore the exciton loss can be attributed to the triplet nonradiative transition process (k_{nr}^{T}). The kinetics parameters were calculated according to the following equations and summarized in Table **S7**.[20, 21]

$$\Phi_{PL} = \Phi_p + \Phi_d \quad (1)$$

$$k_P = \frac{1}{\tau_p} \quad (2)$$

$$k_d = \frac{1}{\tau_d} \quad (3)$$

$$k_r^S = k_p \Phi_p \qquad (4)$$

$$k_{ISC} = k_p(1 - \Phi_p) \qquad (5)$$

$$k_{RISC} = \frac{k_p k_d}{k_{ISC}} \frac{\Phi_d}{\Phi_p} \qquad (6)$$

$$k_{nr}^T = k_d - \Phi_p k_{RISC} \qquad (7)$$

Where the Φ_p and Φ_d are the prompt fluorescent and delayed fluorescent quantum efficiency; k_p is the rate constant of prompt fluorescence; k_d is the rate constant of delayed fluorescence; k_r^S is the radiative decay rate constant of S_1; k_{nr}^T is the non-radiative decay rate constant of T_1; k_{ISC} is the intersystem crossing rate constant; k_{RISC} is the reverse intersystem crossing rate constant.

Table **S2.** Summary of kinetics parameters.

**Compounds**	**Φ_P /%**	**Φ_d /%**	**k_p / 10^8 s^{-1}**	**k_d / 10^4 s^{-1}**	**k_r^S /10^7 s^{-1}**	**k_{nr}^T /10^3 s^{-1}**	**k_{ISC} /10^8 s^{-1}**	**k_{RISC} /10^4 s^{-1}**
**DOBDiKTa**[a]	18	56	3.85	2.33	6.92	7.37	3.15	8.82
**DOBDiKTa**[b]	29	19	5.26	9.09	15.3	66.6	3.74	8.39
**DOBDiKTa**[c]	17	71	3.86	1.67	6.56	2.41	3.20	8.39
**DiKTa**[a]	7	39	2.08	0.41	1.44	2.40	1.94	2.52
**tBuDOBNA**[a]	62	-	1.02	-	-	-	-	-

[a] Measured in spin-coated 1.5 wt% doped thin films in mCP; [b] Measured in toluene solution (1×10^{-5} M); [c] Measured in spin-coated 1.5 wt% doped thin films in mCP/PPT (1:1).

Devices

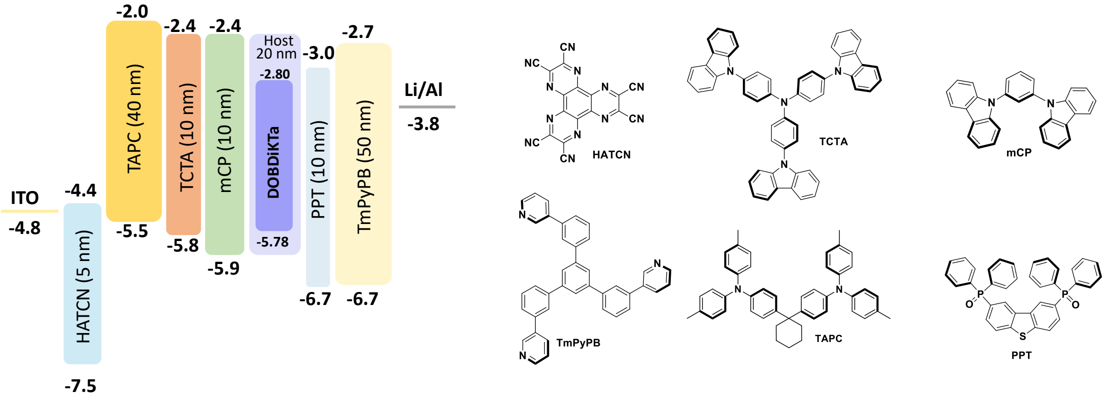

Figure **S29**. Energy level diagram of materials employed in the devices and molecular structure of materials used in the devices.

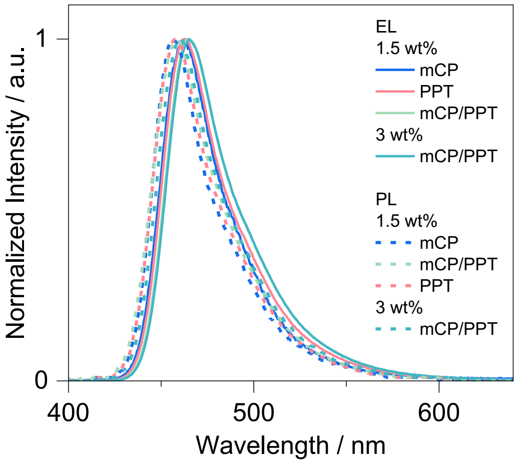

Figure **S30.** Comparation of PL and EL spectrum of **DOBDiKTa.**

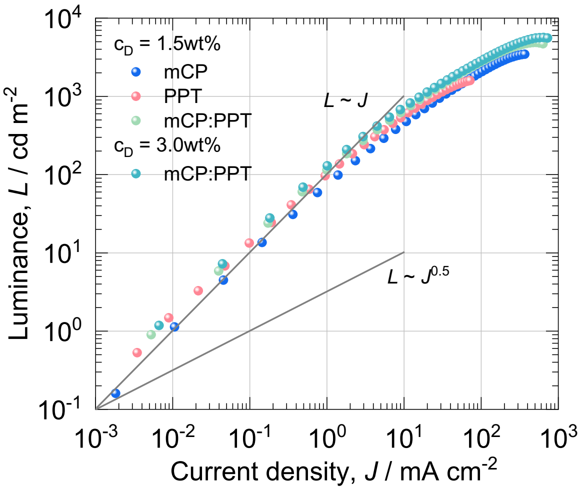

Figure **S31**. Luminance versus current density for the device.

Table **S3**. Key parameters of reported OLED devices based on carbonyl-based MR-TADF OLEDs.

**Emitters**[a]	**λ_{EL} / nm**	**FWHM / nm**	**EQE_{max} / %**	**CIE (x,y)**	**Roll-off / % at EQE_{100}**	**L_{max} / cd m^{-2}**	**Ref.**
**DiKTa**	465	39	14.7	0.14,0.18	43.5	10 385	22
**Mes_3DiKTa**	480	36	21.1	0.12,0.32	31.3	12 949	
**QAD (akaDiKTa)**	468	39	19.4	0.13,0.18	51.5	1100	23
**3Ph-QAD**	480	44	19.1	0.13,0.32	45.5	4975	24
**7Ph-QAD**	472	34	18.7	0.13,0.24	88.7	2944	
**QA-PF**	474	27	16.8	0.12,0.17	66.6	1740	
**QA-PCN**	473	30	16.9	0.12,0.18	44.4	2760	25
**QA-PMO**	484	27	15.0	0.11,0.30	76.6	3040	
**QA-PCZ**	482	29	17.5	0.11,0.28	56.5	3600	
**BOQAO**	484	32	21.8	0.11,.037	-	-	3
**DQAO**	472	34	17.8	0.12,0.18	-	-	26
**QA-2**	463	37	19.0	0.13,0.14	-	-	27
**DOB-DiKTa**	**458**	**38**	**17.4**	**0.14,0.12**	**32.2**	**4707**	**This work**

References

1. H. Lim, H. J. Cheon, S. J. Woo, S. K. Kwon, Y. H. Kim and J. J. Kim, *Adv. Mater.*, 2020, **32**, 2004083.
2. D. Hall, S. M. Suresh, P. L. dos Santos, E. Duda, S. Bagnich, A. Pershin, P. Rajamalli, D. B. Cordes, A. M. Z. Slawin, D. Beljonne, A. Köhler, I. D. W. Samuel, Y. Olivier and E. Zysman-Colman, *Adv. Opt. Mater.*, 2020, **8**, 1901627.
3. Y.-J. Yu, S.-N. Zou, C.-C. Peng, Z.-Q. Feng, Y.-K. Qu, S.-Y. Yang, Z.-Q. Jiang and L.-S. Liao, *J. Mater. Chem. C*, 2022, **10**, 4941-4946.
4. H. B. S. G. W. T. M. J. Frisch, G. E. Scuseria, M. A. Robb, J. R. Cheeseman, G. Scalmani, V. Barone, B. Mennucci, G. A. Petersson, H. Nakatsuji, M. Caricato, X. Li, H. P. Hratchian, A. F. Izmaylov, J. Bloino, G. Zheng, J. L. Sonnenberg, M. Hada, M. Ehara, K. Toyota, R. Fukuda, J. Hasegawa, M. Ishida, T. Nakajima, Y. Honda, O. Kitao, H. Nakai, T. Vreven, J. A. Montgomery Jr., J. E. Peralta, F. Ogliaro, M. Bearpark, J. J. Heyd, E. Brothers, K. N. Kudin, V. N. Staroverov, R. Kobayashi, J. Normand, K. Raghavachari, A. Rendell, J. C. Burant, S. S. Iyengar, J. Tomasi, M. Cossi, N. Rega, J. M. Millam, M. Klene, J. E. Knox, J. B. Cross, V. Bakken, C. Adamo, J. Jaramillo, R. Gomperts, R. E. Stratmann, O. Yazyev, A. J. Austin, R. Cammi, C. Pomelli, J. W. Ochterski, R. L. Martin, K. Morokuma, V. G. Zakrzewski, G. A. Voth, P. Salvador, J. J. Dannenberg, S. Dapprich, A. D. Daniels, Ö. Farkas, J. B. Foresman, J. V. Ortiz, J. Cioslowski, D. J. Fox, , Gaussian 16 Revis, Wallingford, CT, 2016.
5. N. O. Winter and C. Hättig, *J. Chem. Phys*, 2011, **134**, 184101.
6. a. d. o. U. o. K. a. F. TURBOMOLE V7.4 2017 and -. Karlsruhe GmbH, TURBOMOLE GmbH, since S105 2007, 1989-2007.
7. C. Adamo and V. Barone, *J. Chem. Phys.* , 1999, **110**, 6158-6170.
8. T. H. Dunning Jr, *J. Chem. Phys.*, 1989, **90**, 1007-1023.
9. S. Grimme, *Chem. Phys. Lett.*, 1996, **259**, 128-137.
10. S. Hirata and M. Head-Gordon, *Chem. Phys. Lett.*, 1999, **314**, 291-299.
11. G. Petersson and M. A. Al‐Laham, *J. Chem. Phys.*, 1991, **94**, 6081-6090.
12. K. Momma and F. Izumi, *J. Appl. Crystallogr.*, 2011, **44**, 1272-1276.
13. W. Humphrey, A. Dalke and K. Schulten, *J. Mol. Graphics*, 1996, **14**, 33-38.
14. N. G. Connelly and W. E. Geiger, *Chem. Rev.*, 1996, **96**, 877-910.
15. C. M. Cardona, W. Li, A. E. Kaifer, D. Stockdale and G. C. Bazan, *Adv. Mater.*, 2011, **23**, 2367-2371.
16. G. A. D. Crosby, J.N., *J. Phys. Chem*, 1971, **75**, 991-1024.
17. W. H. Melhuish, *J. Chem. Phys.*, 1961, **65**, 229-235.
18. N. C. Greenham, I. D. W. Samuel, G. R. Hayes, R. T. Phillips, Y. A. R. R. Kessener, S. C. Moratti, A. B. Holmes and R. H. Friend, *Chem. Phys. Lett.*, 1995, **241**, 89-96.
19. J. Mooney and P. Kambhampati, *J. Phys. Chem. Lett.* , 2013, **4**, 3316-3318.
20. K. Masui, H. Nakanotani and C. Adachi, *Org. Electron.*, 2013, **14**, 2721-2726.
21. Y. Tsuchiya, S. Diesing, F. Bencheikh, Y. Wada, P. L. Dos Santos, H. Kaji, E. Zysman-Colman, I. D. Samuel and C. Adachi, *J. Phys. Chem. A*, 2021, **125**, 8074-8089.
22. D. Hall, S. M. Suresh, P. L. dos Santos, E. Duda, S. Bagnich, A. Pershin, P. Rajamalli, D. B. Cordes, A. M. Z. Slawin, D. Beljonne, A. Köhler, I. D. W. Samuel, Y. Olivier and E. Zysman-Colman, *Adv. Opt. Mater.*, 2020, **8**, 1901627.
23. Y. Yuan, X. Tang, X.-Y. Du, Y. Hu, Y.-J. Yu, Z.-Q. Jiang, L.-S. Liao and S.-T. Lee, *Adv. Opt. Mater.*, 2019, **7**, 1801536.
24. X. Li, Y.-Z. Shi, K. Wang, M. Zhang, C.-J. Zheng, D.-M. Sun, G.-L. Dai, X.-C. Fan, D.-Q. Wang and W. Liu, *ACS Appl. Mater. Interfaces*, 2019, **11**, 13472-13480.
25. X. Qiu, G. Tian, C. Lin, Y. Pan, X. Ye, B. Wang, D. Ma, D. Hu, Y. Luo and Y. Ma, *Adv. Opt. Mater.*, 2021, **9**, 2001845.
26. S.-N. Zou, C.-C. Peng, S.-Y. Yang, Y.-K. Qu, Y.-J. Yu, X. Chen, Z.-Q. Jiang and L.-S. Liao, *Org. lett.*, 2021, **23**, 958-962.
27. H. Min, I. S. Park and T. Yasuda, *Angew. Chem. Int. Ed.*, 2021, **133**, 7721-7726.
